# Improving cytosolic aspartate biosynthesis increases glucoamylase production in *Aspergillus niger* under oxygen limitation

**DOI:** 10.1186/s12934-020-01340-1

**Published:** 2020-04-03

**Authors:** Weiqiang Cao, Guan Wang, Hongzhong Lu, Liming Ouyang, Ju Chu, Yufei Sui, Yingping Zhuang

**Affiliations:** State Key Laboratory of Bioreactor Engineering, East China University of Science and Technology, Shanghai, People’s Republic of China

**Keywords:** *Aspergillus niger*, Glucoamylase, Limited amino acids, Gene overexpression, Multi-omics study

## Abstract

**Background:**

Glucoamylase is one of the most industrially applied enzymes, produced by *Aspergillus* species, like *Aspergillus niger*. Compared to the traditional ways of process optimization, the metabolic engineering strategies to improve glucoamylase production are relatively scarce.

**Results:**

In the previous study combined multi-omics integrative analysis and amino acid supplementation experiment, we predicted four amino acids (alanine, glutamate, glycine and aspartate) as the limited precursors for glucoamylase production in *A. niger*. To further verify this, five mutants namely OE-ala, OE-glu, OE-gly, OE-asp1 and OE-asp2, derived from the parental strain *A. niger* CBS 513.88, were constructed respectively for the overexpression of five genes responsible for the biosynthesis of the four kinds of amino acids (*An11g02620*, *An04g00990*, *An05g00410*, *An04g06380* and *An16g05570*). Real-time quantitative PCR revealed that all these genes were successfully overexpressed at the mRNA level while the five mutants exhibited different performance in glucoamylase production in shake flask cultivation. Notably, the results demonstrated that mutant OE-asp2 which was constructed for reinforcing cytosolic aspartate synthetic pathway, exhibited significantly increased glucoamylase activity by 23.5% and 60.3% compared to CBS 513.88 in the cultivation of shake flask and the 5 L fermentor, respectively. Compared to *A. niger* CBS 513.88, mutant OE-asp2 has a higher intracellular amino acid pool, in particular, alanine, leucine, glycine and glutamine, while the pool of glutamate was decreased.

**Conclusion:**

Our study combines the target prediction from multi-omics analysis with the experimental validation and proves the possibility of increasing glucoamylase production by enhancing limited amino acid biosynthesis. In short, this systematically conducted study will surely deepen the understanding of resources allocation in cell factory and provide new strategies for the rational design of enzyme production strains.

## Introduction

*Aspergillus niger*, as one of the most efficient cell factories for enzymes, organic acids and food additives production, is widely applied in biochemistry industry. Glucoamylase, especially fungal glucoamylases, has gained great importance because of its wide application in different industries. Therefore, it has been extensively studied during the past few decades [1]. Common strategies to improve glucoamylase production in *A. niger* include fermentation process optimization and genetic engineering. Fermentation process optimization mainly include mycelial morphology control [2], fermentation parameter optimization [3], culture medium optimization [4, 5] and the oxygen limitation strategy [6].

In industrial glucoamylase production, oxygen limitation strategy is widely applied because high glucoamylase yield to carbon source can be achieved and the byproducts are more efficiently reused [6]. In the previous study [7], we conducted the multi-omics integrative analysis of a glucoamylase over-producing strain under oxygen-limited conditions in a 5 L fermentor. Results indicated that under oxygen-limited conditions, cell growth arrest occurs and more precursors such as amino acids may be channeled towards enzyme production, which further increases the enzyme yield. However, we found that alanine, glutamate, aspartate and glycine, whose intracellular pools decreased sharply in oxygen-limited phases, might form restrictions for further improvement of glucoamylase production. Among them, alanine, aspartate and glycine account for high ratios in amino acid composition of glucoamylase (Additional file 1). Besides, exogenous addition of these amino acids in medium also significantly improved glucoamylase production, which was another convincing evidence for our prediction on amino acid bottlenecks [7].

Addition of amino acids in the medium is a common way of increasing the supply of limited amino acids to enhance cell growth as well as production of protein products. In several yeast species such as *P. pastoris*, *P. stipitis* and *S. cerevisiae*, exogenous amino acids are supplemented in the culture media to improve the heterologous protein production [8–10]. However, this strategy is not the solution for industrial processes mainly because of its high costs. Another strategy of elevating amino acid supply to improve protein production is engineering amino acid or tRNA pools. Some pieces of work were reported in *E. coli* and yeast. Xia et al. [11] elevated the glycyl-tRNA pool in *E. coli* by metabolic engineering to enhance the production of a 284.9 kDa recombinant spider silk protein which is extremely rich in glycine. Another similar study was reported by Cao et al. [12]. Glycyl/alanyl-tRNA pool was enhanced in the engineered *E. coli* to achieve high expression of recombinant protein with high molecular weight and Gly-Ala repeated sequence. In *P. pastoris*, Zahrl et al. [13] concluded in their review that the supply of ATP and NADH was possibly elevated by engineering of central carbon metabolism. This benefited the biosynthesis of amino acids and hence elevated the amino acid pools to improve protein production. However, in filamentous fungi, no previous works were reported on the engineering of amino acid pools to improve enzyme production because the regulation of amino acid biosynthesis, as well as the composition and biosynthesis of protein, is rather complex and unclear, which has hampered the development of relevant studies.

Metabolic engineering of amino acid biosynthesis is rather challenging because there exists a so-called metabolic interlock, which means co-regulation between enzymes of different amino acid biosynthetic pathways in fungi [14]. This phenomenon was a derepression of enzymes of different amino acid biosynthetic pathways upon limitations for amino acids, which was firstly described in *Neurospora crassa* [15–17] as cross-pathway control and has been called the “general control of amino acid biosynthesis” in *S. cerevisiae* [18]. In *N. crassa*, Carsiotis et al. [15, 16] reported that enzymes of tryptophan, histidine and arginine biosynthetic pathways are subject to the cross-pathway control. Niederberger et al. [14] found that the general control in *S. cerevisiae* affects biosynthetic pathways of all branched amino acids (aromatic amino acids and the aspartate family) and the pathways for the basic amino acids. For these amino acids, allosteric feedback inhibition, repression of enzymes in common for amino acids of branched pathways and active sequestration of amino acids [19] will cause amino acid imbalances, thus leading to amino acid limitations. General control helps the cell to cope efficiently with such conditions [14].

Genetic engineering strategies were also applied in glucoamylase overproduction, among which one efficient strategy is the introduction of additional copies of the glucoamylase encoding gene *glaA*. Verdoes et al. [20] introduced multi-copies of *glaA* into the genome of a laboratory *A. niger* strain to obtain glucoamylase-overproducing strains. Xin et al. [21] also demonstrated that overexpression of glucoamylase encoding gene *glaA* and α-amylase encoding gene *amyA* substantially increases the expression and secretion of glucoamylase in *A. niger*. Furthermore, researchers were also focused on the improvement of protein secretion ability to increase glucoamylase production [22, 23]. However, introduction of excessive *glaA* copies may overload host cells and fails to increase glucoamylase production. To efficiently improve the glucoamylase yield, cellular resources accumulated in primary metabolism (e.g. amino acids, energy and cofactors) require to be allocated coordinately to cell growth and product biosynthesis.

**Fig. 1:**
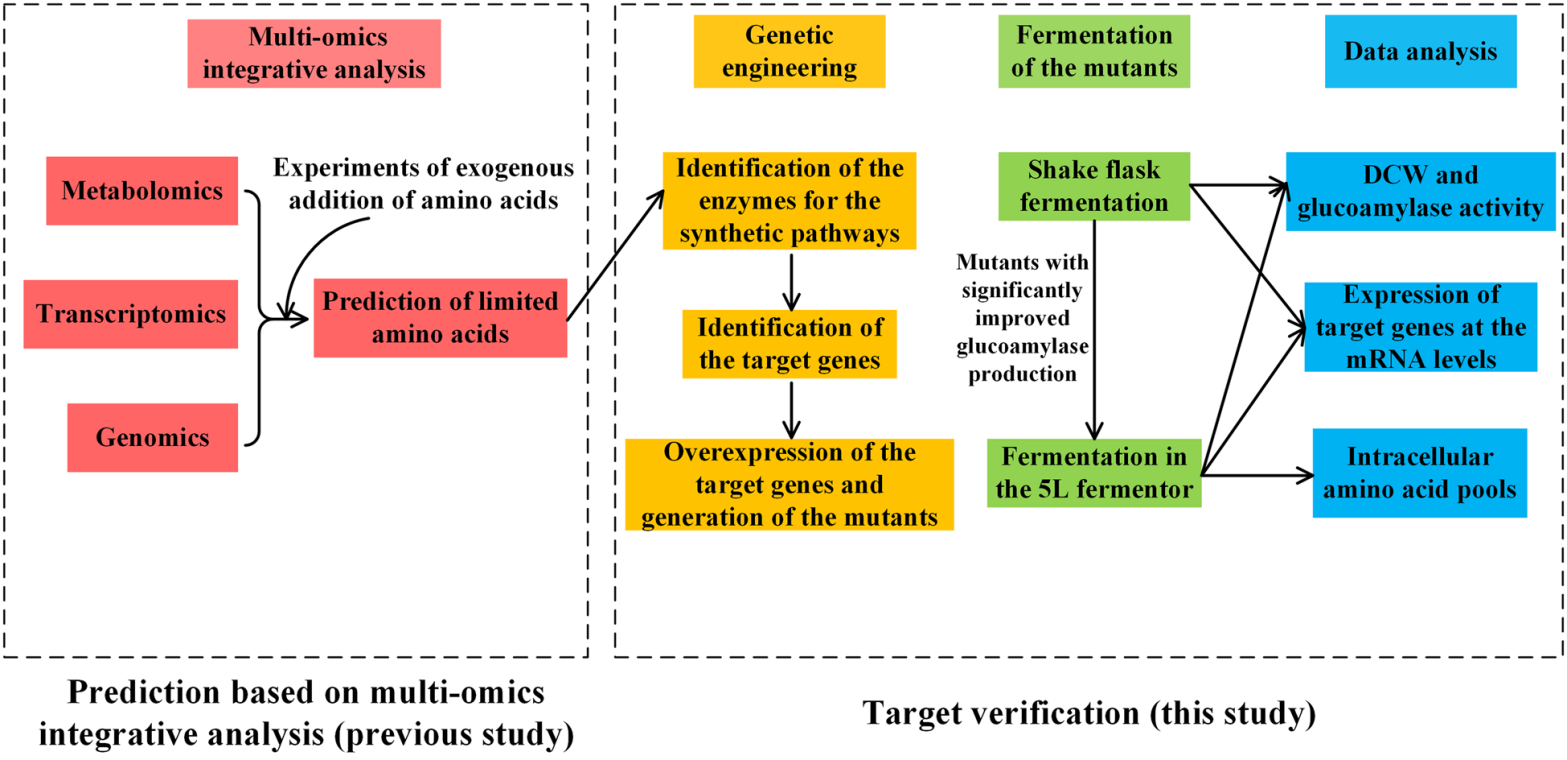
Framework of combining biomarker prediction based on previous multi-omics integrative analysis with verification in this study

This study aims to investigate if the enhancement of the biosynthesis of the four predicted limited amino acids can improve the glucoamylase production in *A. niger* CBS 513.88. The framework of this study is summarized in Fig. 1. Firstly, according to the genome-scale metabolic model iHL1210 [24] and the transcriptomic data obtained in our previous study [7], metabolic enzymes for the biosynthetic pathways of the limited amino acids were identified and the genes involved were overexpressed in *A. niger* CBS 513.88, generating the mutants (Table 1). Secondly, shake flask fermentation was carried out to evaluate the glucoamylase production performance of the mutants. Finally, based on the shake flask fermentation results, mutants with significant production increase were further studied in a 5 L fermentor. Moreover, amino acid pools and transcription levels were measured to elucidate the mechanism of the influence of the identified limited amino acids on glucoamylase production. Here, our study provides a possibility of increasing intracellular limited amino acids to improve protein production in *A. niger*.

**Table 1:** Profiles of the mutant strains constructed in this study

Mutant strains	Gene overexpressed	Enzyme encoded	Pathway	Related limiting amino acids
OE-ala	*An11g02620* (Alanine transaminase)	EC 2.6.1.2	pyr + glu → akg + ala	Alanine
OE-glu	*An04g00990* (Glutamate dehydrogenase, *gdhA*)	EC 1.4.1.4	nh_3_ + akg + h+nadph→h_2_o + glu + nadp	Glutamate
OE-gly	*An05g00410* (Glycine/serine hydroxymethyltransferase)	EC 2.1.2.1	thf + ser → h_2_o + gly+metthf	Glycine
OE-asp1	*An04g06380* (Mitochondrial aspartate aminotransferase)	EC 2.6.1.1	oa(m) + glu(m)→asp(m) + akg(m)	Aspartate
OE-asp2	*An16g05570* (Cytosolic aspartate aminotransferase)	EC 2.6.1.1	oa + glu → asp + akg	Aspartate
CBS-Δ*olvA*	*olvA* deletion strain			

*pyr* pyruvate, *glu*, glutamate, *akg* 2-Oxoglutarate, *ala* alanine, *thf* tetrahydrofolate, *ser* serine, *gly* glycine, *metthf* 5,10-Methylenetetrahydrofolate, *oa* oxaloacetate, *asp* aspartate. *m* (mitochondrion)

## Materials and methods

### Strains

The model glucoamylase-producing strain *Aspergillus niger* CBS 513.88 was used as the parental strain. All the mutants were obtained by protoplasts mediated transformation [25] from *A. niger* CBS 513.88. We selected the pigment gene *olvA* in the genome of *A. niger* CBS 513.88 as the integration site of the expression cassette. Gene *olvA* is required for development of the characteristic black spore pigmentation of *A. niger* [26]. *A. niger* OlvA protein is highly homologous to the *A. fumigatus* AygA protein, which has been shown to convert the heptaketide naphthopyrone (formed by the polyketide synthase) to the pentaketide 1,3,6,8-tetrahydroxynaphthalene (T4HN) [27]. Interruption of *olvA* leads to the color change of spores from black to olive [26] (Additional file 2: Fig. S1), which brings great convenience to pre-screening of the transformants.

### Media and culture conditions

Luria Broth (LB) culture medium supplemented with 100 μg/ml of ampicillin and culture conditions of 37 °C and 220 rpm were used for *E. coli* cultivation. Potato dextrose agar (PDA) medium and culture conditions of 30 °C were used for *A. niger* plate cultivation. Potato dextrose agar (PDA) medium supplemented with 100 μg/ml of hygromycin and culture conditions of 30 °C were used for isolation and purification of the mutants.

### Constructions of donor DNA for *olvA* deletion and target gene overexpression

All the primers used in this section were listed in Additional file 3: Table S1. All the plasmids were produced in *Escherichia coli* DH5α cells. All the procedures were performed according to standard methods or the manufacturer’s instructions. Donor DNA is required for CRSIPR-Cas9 mediated *olvA* deletion and target gene overexpression by homologous recombination. (Additional file 2: Fig. S2) exhibits the process of donor DNA construction. For the construction of donor DNA for *olvA* deletion, the hygromycin expression cassette (P*gpdA*+*hygR*+T*trpC*) was obtained by PCR amplification from the fungal expression plasmid pAN 7-1 (Additional file 3: Table S4) and then was flanked by 1 kb homologous arm (HA) of *olvA*. For the construction of donor DNA for target gene overexpression, recombinant plasmids were firstly constructed. The backbone of the recombinant plasmids was obtained by PCR amplification from pAN 7-1 excluding the hygromycin gene. The candidate genes were amplified by PCR from the genome of CBS 513.88 and cloned into the backbone by ClonExpressII One Step Cloning Kit (Vazyme) separately, generating the recombinant plasmids for overexpression of the candidate genes. After construction, the hygromycin gene in original pAN 7-1 was replaced by candidate gene so the gene was under the control of the constitutive strong promoter P*gpdA*. Afterwards, the expression cassette of the candidate gene (P*gpdA*+candidate gene+T*trpC*) was obtained by PCR amplification from the recombinant plasmid and then was flanked by 1 kb homologous arm (HA) of *olvA*.

### CRISPR/Cas9 mediated *olvA* deletion and target gene overexpression by homologous recombination

Protoplast-mediated transformation method is used for generating transformants. The generic process of mutant generation was shown in (Additional file 2: Fig. S3). We used the recombinant plasmid pAN-*olvA* (constructed in our lab, Additional file 3: Table S4) to express the selectable marker *hygR* for hygromycin, together with Cas9 and sgRNA for *olvA*. For *olvA* deletion, pAN-*olvA* and donor DNA for *olvA* deletion (Additional file 2: Fig. S2) were co-transformed into *A. niger* protoplasts. For gene overexpression, pAN-*olvA* and donor DNA for gene overexpression (Additional file 2: Fig. S2) were co-transformed into *A. niger* protoplasts. The donor DNA, after integration by homologous recombination, would then introduce the expression cassette (P*gpdA*+*hygR* or candidate gene+T*trpC*) at the location of *olvA*. Regeneration medium supplemented with 150 μg/ml of hygromycin was used to generate selection pressure for the plasmid. The transformants, whose spores with olive color, were picked out for further single colony separation. PDA medium supplemented with 100 μg/ml of hygromycin was used for isolation and purification for the single colony. After genome extraction and PCR verification with three pairs of primers (P1/P2, P5/P6 and P1/P3 (Additional file 3: Table S2)) of the single colony, positive mutants were successfully screened out.

### Shake flask and the 5 L fermentor cultivation of *A. niger*

#### Seed culture

100 ml seed culture medium (20 g/l glucose, 20 g/l corn steep liquor) was inoculated with 10^6^/ml of washed spores in 500 ml shake flasks and cultured under the conditions of 34 °C, 150 rpm for 24 h.

#### Fermentation in 500 ml shake flask

The fermentation medium (g/l: glucose 50, CaCl_2_ 0.076, MgSO_4_·7H_2_O 1, EDTA-2Na 0.67, NaNO_3_ 7.5, KH_2_PO_4_ 3, NaH_2_PO_4_·2H_2_O 1.69, MnSO_4_·H_2_O 0.04, ZnCl_2_ 0.02, CuSO_4_·5H_2_O 0.015, CoCl_2_·6H_2_O 0.015, FeSO_4_·7H_2_O 0.3, 0.1% antifoam reagent, initial pH = 5.5, 5 glass beads with diameter of 3 mm) was inoculated with 3 ml seed culture broth (pre-grown mycelia) and cultured under the conditions of 34 °C, 170 rpm.

#### Fed-batch fermentation in the 5 L fermentor

3.5 L fermentation medium (g/l: glucose 50, CaCl_2_ 0.076, MgSO_4_·7H_2_O 1, EDTA-2Na 0.67, NaNO_3_ 7.5, KH_2_PO_4_ 3, NaH_2_PO_4_·2H_2_O 1.69, MnSO_4_·H_2_O 0.04, ZnCl_2_ 0.02, CuSO_4_·5H_2_O 0.015, CoCl_2_·6H_2_O 0.015, FeSO_4_·7H_2_O 0.3, 0.1% antifoam reagent, initial pH = 4.5) was inoculated with 200 ml seed culture broth (pre-grown mycelia). The agitation and aeration were 375 rpm and 1 vvm, respectively. The pressure and temperature were 0.05 MPa and 34 °C, respectively. The fermentation pH was controlled at 4.5 by 5 M NaOH. When glucose concentration was decreased to around 10 g/l, supplemented medium (glucose 200 g/l and all the other components in the fed-batch reservoir are the same as those in the batch medium) was fed into the fermentor after batch culture. The feeding rate was initially calculated based on the glucose consumption rate in batch culture before feeding and was then adjusted based on the residual glucose in the broth to control the glucose concentration between 5 to 10 g/l.

### Dry cell weight (DCW) and enzyme activity assay

10 ml fermentation broth was filtered by pre-dried and pre-weighed filter paper. The filtered biomass, washed twice by deionized water to remove other solutes, was then dried in 80 °C for 24 h. The dried biomass should be cooled in a desiccator and then weighed immediately.

The enzyme activity of glucoamylase is expressed in AGI units (Amyloglucosidase). One AGI unit is defined as the amount of enzyme that produces 1 μmol of glucose per minute at pH 4.3 and at 60 °C from a soluble starch substrate. The glucoamylase activity was determined at 37 °C and pH 4.30 using the colorless chromophore p-nitrophenyl-α-d-glucopyranoside (pNP-G) as substrate. 230 μl of 2 g/l pNP-G (pre-warmed in 37 °C for 5 min) was mixed with 20 μl supernatant of the fermentation broth. 100 μl of 3 M Na_2_CO_3_ was added after an incubation in 37 °C water bath for 20 min. Enzyme activity was determined by the absorbance of the mixed solution at the wavelength of 405 nm. Before measuring samples, we established the standard curve of enzyme activity (R^2^ > 0.999): enzyme activity = dilution ratio × (OD_405_ + 0.01)/0.008.

### RNA extraction and real-time quantitative PCR

For RNA extraction, 1 ml fermentation samples were collected. For shake flask fermentation, samples were taken at 36 h (exponential growth phase) and 72 h (oxygen-limited phase). For fed-batch fermentation in the 5 L fermentor, samples were taken at 36 h (exponential growth phase in batch culture) and 72 h (oxygen-limited phase in feeding stage). All procedures were performed according to the manufacturer’s instructions. Fungal Total RNA Isolation Kit (Sangon Biotech) was used for RNA extraction after liquid nitrogen grinding. Total RNA was reverse-transcribed by PrimeScript^™^ RT reagent kit with gDNA Eraser (Perfect Real Time, Takara). PCR was performed by using TB Green *Premix Ex taq*^*™*^ II (Takara) and Bio-rad CFX96 real-time PCR Detection system. The conditions were used: 95 °C for 30 s, followed by 39 cycles of 95 °C for 5 s and 60 °C for 30 s. The primers used in this section were listed in Additional file 3: Table S3.

### Sampling and intracellular amino acid pools determination

A rapid sampling device was used to take about 1 g (precisely determined) broth from the 5 L fermentor into 10 ml precooled − 30 °C 40% methanol (v/v). After filtered and washed by 15 ml precooled − 30 °C 40% methanol (v/v), the filter cake was thrown into 25 ml of 75 °C pre-warmed 75% ethanol (v/v), with the addition of 100 μl ^13^C internal standard solution for extraction in 95 °C water bath for 3 min [28]. The extracted solution was concentrated to 0.6 g after cooling and 100 μl was used for freeze drying. After that, 75 μl acetonitrile (dissolving agent) and 75 μl MTBSTFA+ 1% TBDMSCl (derivating agent) were added respectively for reaction in 70 °C for 1 h. Then the samples were centrifuged at 10,000*g* for 2 min and the supernatant was collected for amino acids detection by GC–MS (Agilent, Santa Clara, CA, USA).

### Statistical analysis

Unless otherwise noted, all experiments were performed in three replicates and statistical significance was determined by two-tailed Student’s *t* test.

## Results

### Characterization of the five overexpression mutants

Five genes encoding the enzymes for the biosynthesis of four kinds of probable limiting amino acids, namely alanine transaminase, glutamate dehydrogenase, glycine/serine hydroxymethyltransferase, mitochondrial aspartate aminotransferase and cytosolic aspartate aminotransferase were overexpressed in the *olvA* locus under the control of the constitutive strong promoter P*gpdA*. In addition, an *olvA* deletion strain CBS-Δ*olvA* derived from *A. niger* CBS 513.88 was also generated as the control. The detailed information of the mutants was shown in Table 1.

### Cell growth and enzyme producing features of the constructed mutants in shake flask cultivation

**Fig. 2:**
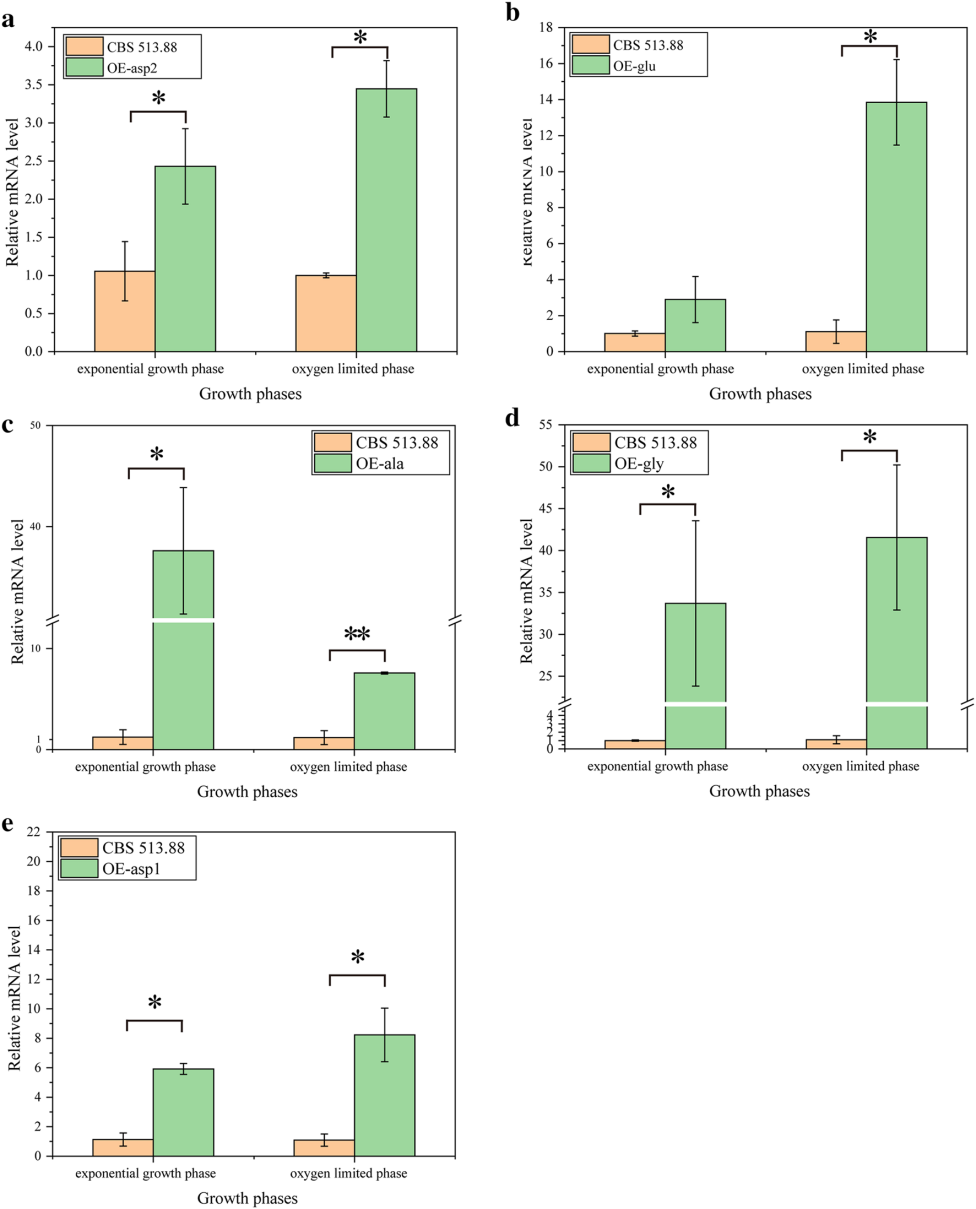
Relative mRNA levels of genes *An16g05570***a**, *An04g00990***b**, *An11g02620***c**, *An05g00410***d** and *An04g06380***e** in the corresponding mutants OE-asp2 **a**, OE-glu **b** OE-ala **c**, OE-gly **d** and OE-asp1 **e**, respectively. Samples were taken at 36 h (exponential phase) and 72 h (oxygen-limited phase). Data represent the average values and standard deviations from three replicates. Statistical significance was performed using a two-tailed Student’s *t* test (*p < 0.05, **p < 0.01)

The glucoamylase activity and dry cell weight (DCW) in shake flask cultivation were measured to evaluate the yield of the mutants. First of all, mutant CBS-Δ*olvA* showed no significant difference in glucoamylase production and biomass growth compared with CBS 513.88 during the fermentation (Additional file 2: Fig. S4a, b), indicating that gene *olvA* is an ideal locus for the integration of overexpression cassettes. As shown in Fig. 2a, the relative mRNA level of gene *An16g05570* in mutant OE-asp2 was approximately 2.5-fold and 3.5-fold higher than that of the parental strain CBS 513.88 during exponential phase and oxygen limited phase, respectively. Similarly, the relative mRNA levels of genes *An04g00990*, *An11g02620*, *An05g00410* and *An04g06380* in the respective mutants OE-glu, OE-ala, OE-gly and OE-asp1 were significantly higher than those of the parental strain CBS 513.88 in the two phases (Fig. 2b–e). Collectively, these results verified that the insertion of the target gene overexpression cassettes successfully improved the transcriptional level of the target genes.

**Fig. 3:**
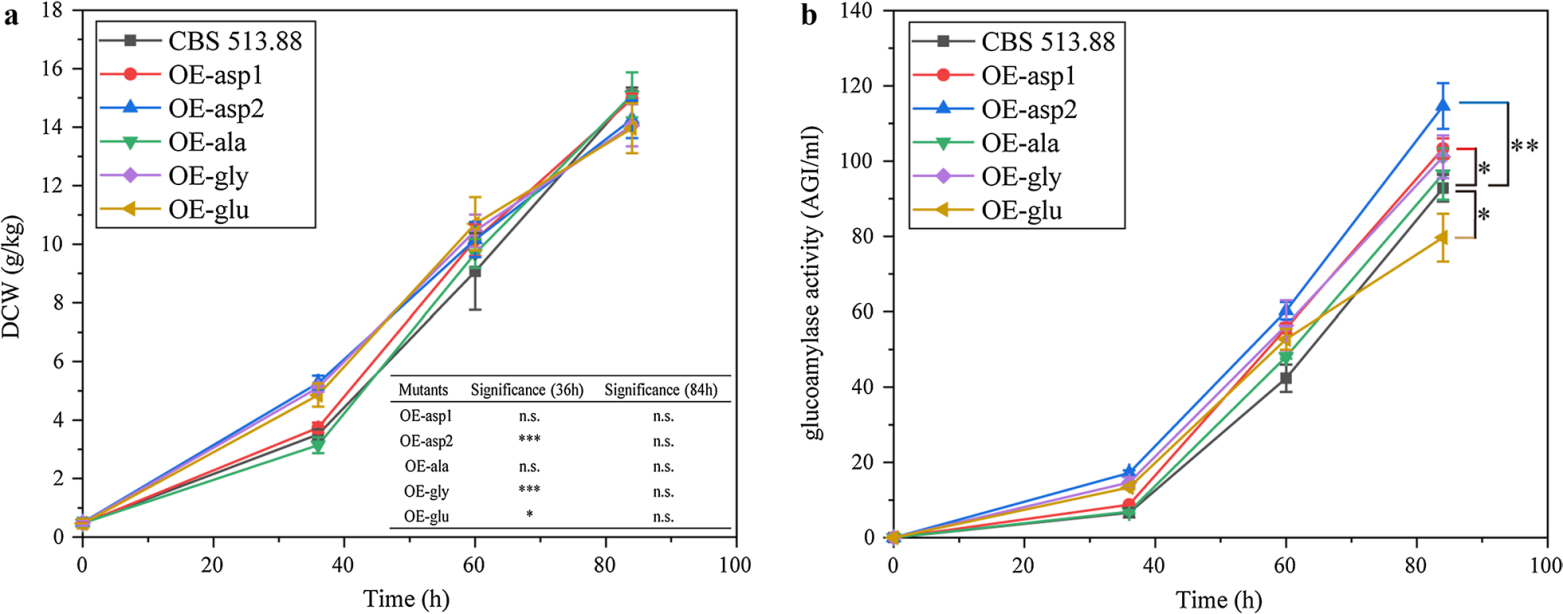
Growth **a** and glucoamylase production **b** profiles of the five mutants and the parental strain CBS 513.88 in shake flask fermentation. Data represent the average values and standard deviations from three replicates. Statistical significance was performed using a two-tailed Student’s *t* test to compare the DCW and glucoamylase activity differences between *A. niger* CBS 513.88 and each of the five mutants in certain time points (***p < 0.001; **p < 0.01; *p < 0.05; n.s., not significant)

Fermentation in the shake flasks revealed that mutants OE-asp2, OE-gly and OE-glu exhibited a significant higher DCW at the time point of 36 h compared to CBS 513.88 (Fig. 3a). However, all the five mutants and CBS 513.88 finally reached the biomass concentration at the value of 14–15 g DCW/kg, which showed no significant difference (Fig. 3a). Mutant OE-asp2 demonstrated 114.66 AGI/ml of glucoamylase activity, a yield of 0.161 g glucoamylase per g DCW (1 mg glucoamylase equals to 50 AGI units of enzyme activity), which was 23.5% higher than that of the parental strain CBS 513.88 (92.81 AGI/ml) while mutant OE-asp1 displayed 103.38 AGI/ml of glucoamylase activity, a yield of 0.138 g glucoamylase per g DCW, which was slightly higher than that of CBS 513.88 (Fig. 3b). Conversely, mutant OE-glu produced 79.69 AGI/ml of glucoamylase, a yield of 0.114 g glucoamylase per g DCW, which was decreased compared to CBS 513.88 (Fig. 3b).

### Fed-batch fermentation of the mutants OE-asp1 and OE-asp2 in the 5 L fermentor

The above stated preliminary result revealed that the performance of five mutants in DCW and glucoamylase activity were different in shake flask cultivation. Notably, mutant OE-asp2 exhibited significant increase in glucoamylase production while mutant OE-asp1 also showed a slight increase. Therefore, we focused on these two mutants, raising the question that whether the increase of intracellular aspartate pool resulted from *An04g06380* and *An16g05570* overexpression indeed led to the improvement of glucoamylase production. In order to gain a deeper understanding, fed-batch fermentation in the 5 L fermentor was carried out and the results were shown in (Additional file 2: Fig. S5). After 10 h of lag phase, the growth of the three strains entered into exponential phase with sufficient oxygen and nutrition supply (Additional file 2: Fig. S5a), especially mutant OE-asp2 with a higher specific growth rate (μ) of 0.11 h^−1^ (Table 2). In this phase, there existed no significant difference in glucoamylase production in terms of q_GA_ and Y_p/x_ between the two mutants and *A. niger* CBS 513.88 (Table 2). After about 40 h, the fermentation entered into oxygen-limited phase and supplemented medium was fed into the fermentor. The growth of the three strains slowed down and finally entered stationary phase at about 70 h, reaching a DCW around 15 g/kg (Additional file 2: Fig. S5a). In oxygen-limited phase, the glucoamylase activity of mutant OE-asp2 increased sharply and reached 234 AGI/ml with a yield of 0.61 g glucoamylase per g DCW (twice the yield of CBS 513.88, Table 2), which was 60.3% higher than that of CBS 513.88 (146 AGI/ml) (Additional file 2: Fig. S5b). On the other hand, mutant OE-asp1 showed similar performance of glucoamylase production compared with *A. niger* CBS 513.88 in oxygen-limited phase (Additional file 2: Fig. S5b and Table 2).

**Table 2:** Kinetic parameters of mutant OE-asp2, mutant OE-asp1 and the parental strain CBS 513.88 in the 5 L fed-batch fermentation

	Strains	OE-asp1	OE-asp2	CBS 513.88
Batch phase	μ (h^−1^)	0.062 ± 0.003	0.11 ± 0.02	0.07 ± 0.008
	q_GA_ (mg gDCW^−1^ h^−1^)	7.16 ± 0.01	7.10 ± 0.78	6.75 ± 0.13
	Y_p/x_ (g/g)	0.11 ± 0.006	0.09 ± 0.008	0.10 ± 0.009
Oxygen-limited phase	μ (h^−1^)	0.012 ± 0.001	0.009 ± 0.002	0.013 ± 0.002
	q_GA_ (mg gDCW^−1^ h^−1^)	3.32 ± 0.07	5.56 ± 0.32	3.45 ± 0.15
	Y_p/x_ (g/g)	0.29 ± 0.02	0.61 ± 0.15	0.29 ± 0.02

In batch and oxygen-limited phases, μ and q_GA_ both represent the average values. Data in the table represent the average values and standard deviations from three replicates

*μ* specific growth rate. *q*_*GA*_ specific glucoamylase production rate, *Y*_*p/x*_ yield of glucoamylase based on biomass

### Measurement of transcriptional level and amino acid pools of mutants OE-asp1 and OE-asp2 fermentation in the 5 L fermentor

To confirm the improvement of aspartate biosynthesis at transcriptional level and metabolite level, we further measured the target gene expression and intracellular amino acid pools of the two mutants and *A.niger* CBS 513.88 during fermentation in the 5 L fermentor. As shown in (Additional file 2: Fig. S6), the relative mRNA level of gene *An04g06380* in mutant OE-asp1 was approximately 16-fold and eightfold higher than that of the parental strain CBS 513.88 in exponential and oxygen limited phases, respectively, while the relative mRNA level of gene *An16g05570* in mutant OE-asp2 was approximately fourfold higher than that of CBS 513.88 in both two phases. Taken together, these results suggested that the insertion of overexpression cassettes of genes *An04g06380* and *An16g05570* was able to improve the transcription of the two target genes.

**Fig. 4:**
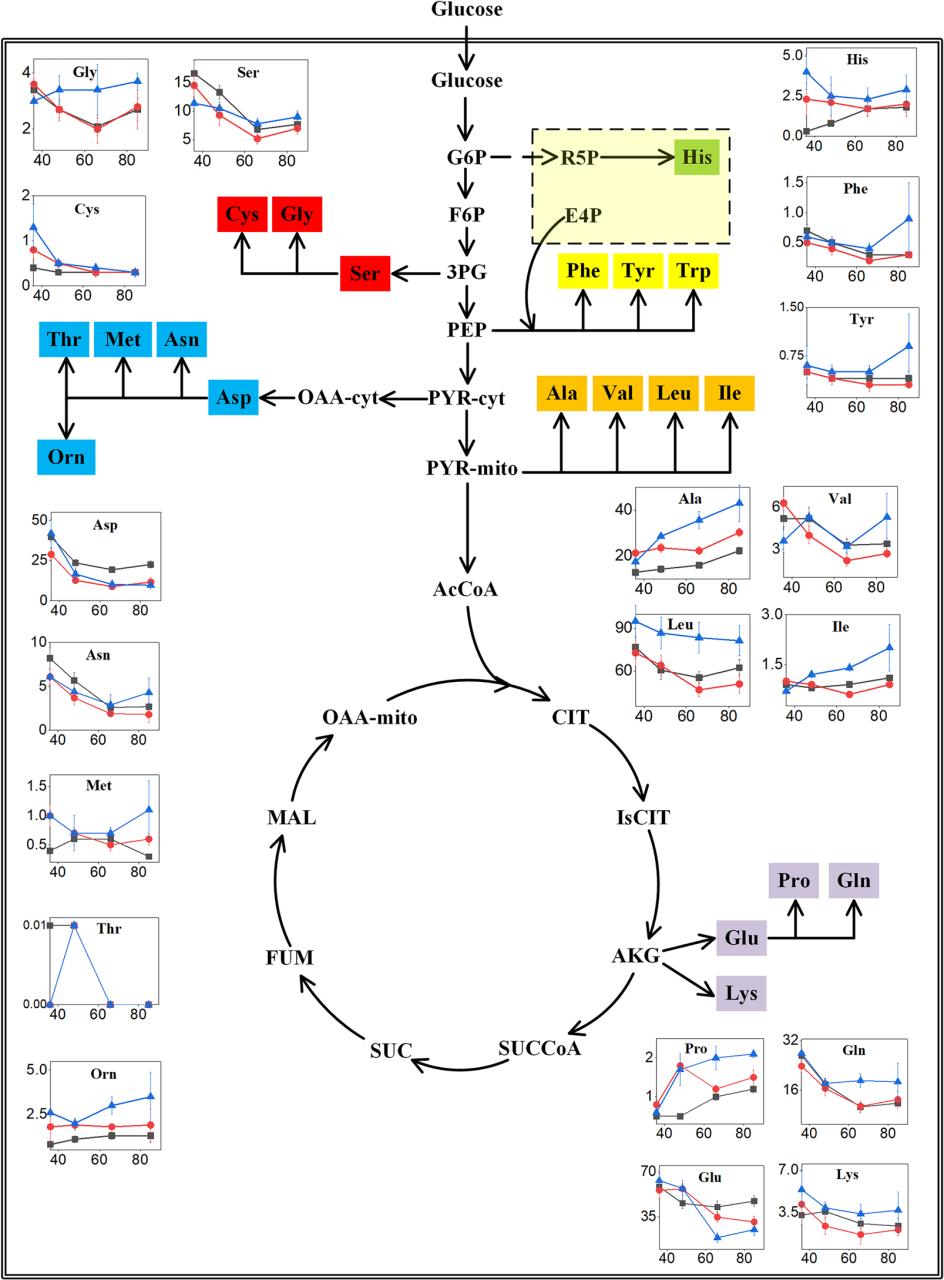
Intracellular amino acid pools of the parental strain CBS 513.88 (black line), mutant OE-asp1 (red line) and mutant OE-asp2 (blue line) at different time points: 36 h (exponential growth phase), 48 h (transition from batch phase to oxygen-limited phase), 66 h (oxygen-limited phase) and 85 h (oxygen-limited phase) in the 5 L fermentor. Data represent the average values and standard deviations from three individual experiments. The X-axis represents the fermentation time (h). The Y-axis represents the intracellular pools of amino acids (μmol/g DCW). The light yellow dotted box represents the Pentose Phosphate pathway that is not showed in detail. *G6P* glucose-6-phosphate, *R5P* ribose-5-phosphate, *E4P* erythrose-4-phosphate, *F6P* fructose-6-phosphate, *3PG* 3-phosphoglycerate, *PEP* phosphoenolpyruvate, *PYR-cyt* cytosolic pyruvate, *PYR-mito* mitochondrial pyruvate, *AcCoA* acetyl-CoA, *CIT* citrate, *IsCIT* isocitrate, *AKG* 2-oxoglutarate, *SUCCoA* succinyl-CoA, *SUC* succinate, *FUM* fumarate, *MAL* malate, *OAA-cyt* cytosolic oxaloacetate, *OAA-mito* mitochondrial oxaloacetate, *His* histidine, *Phe* phenylalanine, *Tyr* tyrosine, *Trp* tryptophan, *Ala* alanine, *Val* valine, *Leu* leucine, *Ile* isoleucine, *Glu* glutamate, *Lys* lysine, *Pro* proline, *Gln* glutamine, *Ser* serine, *Gly* glycine, *Cys* cysteine, *Asp* aspartate, *Asn* asparagine, *Met* methionine, *Thr* threonine, *Orn* ornithine

Furthermore, to demonstrate the amino acid supply in the two mutants, we determined the intracellular amino acid pools of the two mutants and the parental strain CBS 513.88 (Fig. 4). It can be inferred from Fig. 4 that the intracellular amino acid pools of the two mutants differed from those of *A. niger* CBS 513.88 in general and mutant OE-asp2 exhibited more pronounced difference. In mutant OE-asp2, the pools of most amino acids were increased while that of glutamate was decreased and those of amino acids of aspartate family were unaltered. Notably, the pools of alanine, glycine, leucine and glutamine in mutant OE-asp2 were dramatically higher than those of mutant OE-asp1 and *A. niger* CBS 513.88, of which alanine, leucine and glycine account for high ratios in amino acid composition of glucoamylase (Additional file 1). This may contribute to the significant improvement of glucoamylase production in mutant OE-asp2.

## Discussion

### Effects of improving cytosolic aspartate biosynthesis on intracellular amino acid pools and explanations for glucoamylase production increase in mutant OE-asp2

In mutant OE-asp2, the aspartate synthetic pathway: oxaloacetate + glutamate → aspartate + 2-Oxoglutarate was reinforced so more oxaloacetate was consumed, which may result in the increasing supply of pyruvate to synthesize more oxaloacetate. Besides, oxygen limitation will increase the fluxes of EMP pathway in fungi [7, 29, 30], which leads to the accumulation of important precursors (e.g. pyruvate) for amino acid biosynthesis as well. Therefore, the biosynthesis of amino acids of pyruvate family (alanine, valine, leucine and isoleucine) and glycine might be elevated, thus leading to the higher pools of these amino acids in mutant OE-asp2 compared to those in mutant OE-asp1 and *A. niger* CBS 513.88 (Fig. 4). It was indicated that improving cytosolic aspartate biosynthesis had great influence on the biosynthesis of many other amino acids under oxygen-limited conditions, of which alanine, glycine and leucine possess high contents in glucoamylase (Additional file 1).

On the other hand, more glutamate was consumed due to the reinforced aspartate biosynthetic pathway in mutant OE-asp2, leading to the substantially decreased pool of glutamate (Fig. 4). Glutamate serves as the important provider of amino donor in transamination, which plays a primary role in the biosynthesis of amino acids. Therefore, the decreased pool of glutamate will lead to the imbalances of amino acid supply and hence cause the amino acid limitation. The limitation for any amino acid involved in the cross-pathway, or general amino acid control observed in yeast and other fungi will increase the levels of the mRNAs encoding enzymes in most amino acid biosynthetic pathways, which was induced by the transcriptional activator Gcn4p [31]. The biosynthesis of the aromatic amino acids tryptophan, phenylalanine and tyrosine is one of the most well studied amino acid biosynthetic pathways in fungi. In *S. cerevisiae*, at the transcriptional level, expression of most structural genes of aromatic amino acid biosynthetic pathway are regulated by the general amino acid control while at the enzyme level, the carbon flow is controlled mainly by feedback action of the three aromatic amino acid end products [32]. Hinnebusch et al. [33] reported that in *S. cerevisiae*, the expression of the genes involved in the biosynthetic pathways of leucine, isoleucine and glutamine was subject to pathway-specific regulation in addition to the general amino acid control at the transcriptional or translational level. In mutant OE-asp2, integrative action of these amino acid biosynthetic enzymes which are subject to the dual regulation finally enhanced the biosynthesis of these amino acids, thus elevating their intracellular pools.

Taken together, it was indicated that the altered pools of aspartate and glutamate, which directly resulted from the overexpression of *An16g05570* in mutant OE-asp2, led to the increase of most amino acid pools (Fig. 4), possibly due to the increased fluxes of EMP pathway and the regulation of amino acid biosynthesis. Among them, some serve as important amino acid precursors for glucoamylase production, which contributes much to production improvement.

### Possible explanations for different performance of glucoamylase production between mutant OE-asp2 and mutant OE-asp1

**Fig. 5:**
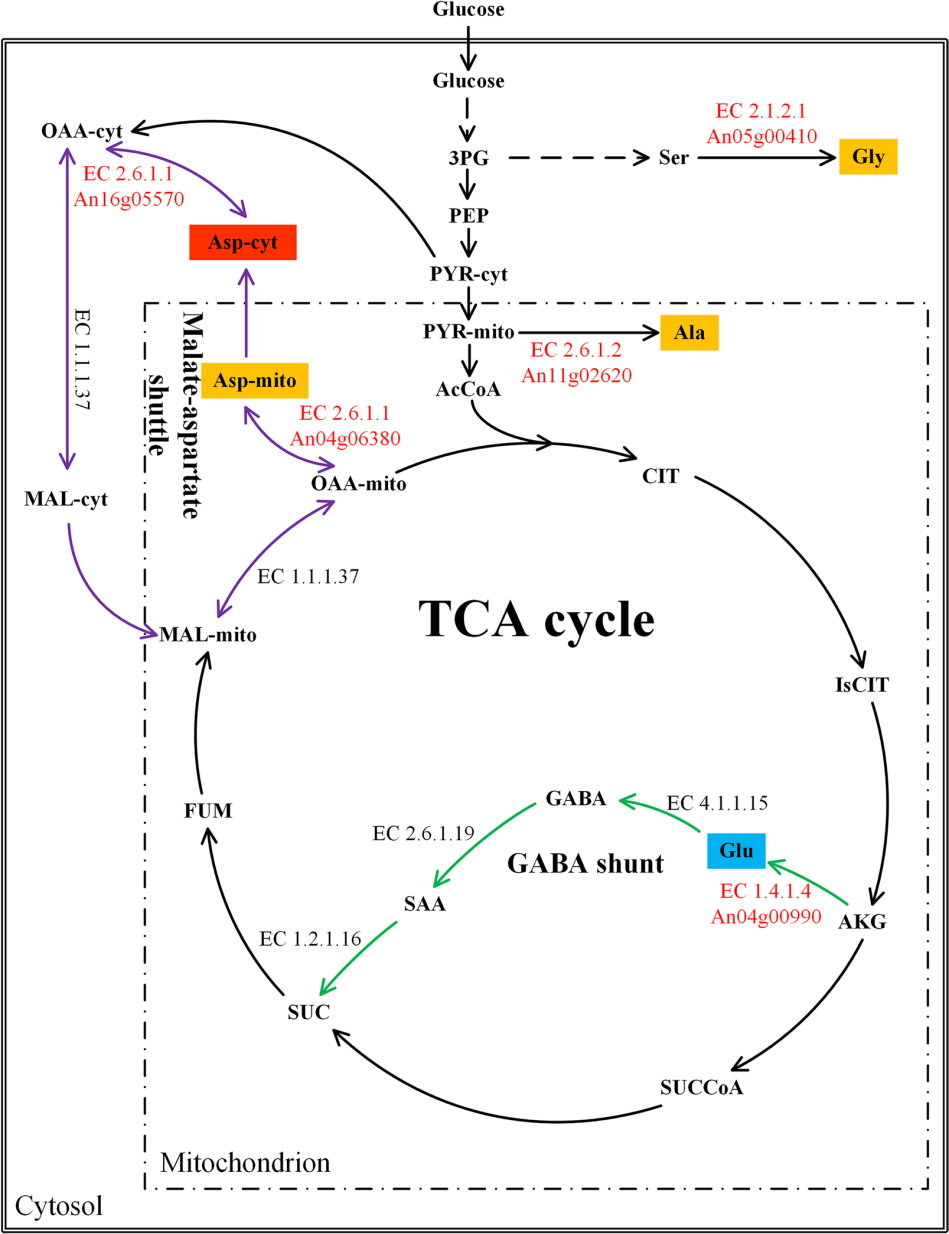
Schematic diagram of TCA cycle, GABA shunt and malate-aspartate shuttle. The biosynthetic pathways of the amino acids with different color backgrounds were reinforced by gene overexpression, leading to different performance of glucoamylase production. The metabolic enzymes (EC numbers) and the encoding genes overexpressed in this study are shown in red. Amino acids in different color backgrounds represent the different glucoamylase production performance of the corresponding overexpression mutants (Table 1) in shake flasks (Red background means the reinforcement of synthetic pathway of this amino acid increased the glucoamylase production in the corresponding mutant compared to CBS 513.88; blue background means glucoamylase production decreased in the corresponding mutant; orange background means the corresponding mutants perform similar glucoamylase production with CBS 513.88). *3PG* 3-phosphoglycerate, *PEP* phosphoenolpyruvate. *PYR-cyt* cytosolic pyruvate, *PYR-mito* mitochondrial pyruvate, *AcCoA* acetyl-CoA, *CIT* citrate, *IsCIT* isocitrate, *AKG* 2-oxoglutarate. *SUCCoA* succinyl-CoA, *SUC* succinate, *FUM* fumarate, *MAL-mito* mitochondrial malate, *OAA-mito* mitochondrial oxaloacetate, *OAA-cyt* cytosolic oxaloacetate, *MAL-cyt* cytosolic malate, *GABA* 4-Aminobutanoate, *SAA* Succinate-semialdehyde, *Ala* alanine, *Glu* glutamate, *Ser* serine, *Gly* glycine, *Asp-cyt* cytosolic aspartate, *Asp-mito* mitochondrial aspartate

Furthermore, the results illustrated an interesting finding that mutant OE-asp1 performed similarly as the parental strain CBS 513.88, significantly distinguished from mutant OE-asp2 in glucoamylase production. The genes *An04g06380* and *An16g05570* encode the isoenzymes named mitochondrial and cytosolic aspartate aminotransferase, respectively, which function in the malate-aspartate shuttle (Fig. 5). In *S. cerevisiae*, these two genes play key roles in the metabolism of amino acids [34]. Mitochondrial and cytosolic aspartate aminotransferase catalyzes the reversible conversion of glutamate and oxaloacetate into 2-Oxoglutarate and aspartate [34]. We speculated that the possible reason may be explained as follows: Firstly, although gene *An04g06380* was overexpressed in mutant OE-asp1, the enzyme activity of mitochondrial aspartate aminotransferase might not be high. Secondly, the oxaloacetate in mitochondrion is probably mainly used for TCA cycle rather than for the engineered pathway: oxaloacetate + glutamate → aspartate + 2-Oxoglutarate. Furthermore, the aspartate which was synthesized in the mitochondrion needs to be transported to cytosol via the aspartate-glutamate exchanger for enzyme production. In mutant OE-asp2, overexpression of *An16g05570* which improved the biosynthesis of aspartate in cytosol can directly take part in the biosynthesis of protein products in cytosolic ribosomes, which resulted in a significant increase of glucoamylase production.

### Possible explanations for decreased glucoamylase production in mutant OE-glu

Throughout the study, the five mutants performed different results in shake flask cultivation. As predicted, mutant OE-asp2 significantly increased glucoamylase production. Most surprisingly, mutant OE-glu showed slight decrease in glucoamylase production, contrary to our prediction. The reaction: nh_3_ + 2-Oxoglutarate + h + nadph → h_2_o + glutamate + nadp, catalyzed by the enzyme EC 1.4.1.4, serves as the first step of GABA shunt. The GABA shunt has been found to be conserved in bacteria and fungi, though there are minor variants in some species [35]. It starts from 2-Oxoglutarate to succinate, two intermediates of TCA cycle, bypassing two reactions of TCA cycle (Fig. 5). The reinforcement of the first reaction of GABA shunt by overexpression of gene *An04g00990* brings more flux into GABA shunt, which is an energetically less efficient pathway than the direct oxidation of 2-Oxoglutarate to succinate by the TCA cycle in fungi [36]. Biosynthesis of amino acids requires large amounts of energy and low energy efficiency may affect the energy supply for the biosynthesis of amino acids. What’s more, we noticed that more NADPH was consumed in the above reinforced pathway, which is also essential in the biosynthesis of other amino acids. Therefore, biosynthesis of other amino acid precursors might be greatly affected in mutant OE-glu, which possibly led to the decrease of glucoamylase production.

### Possible explanations for unaltered glucoamylase production in mutants OE-gly and OE-ala

Just like mutant OE-glu, mutants OE-gly and OE-ala did not improve the glucoamylase production in shake flask fermentation as well. Serine/Glycine hydroxymethyltransferase (SHMT) is an important enzyme of glycine metabolism, which catalyses the reversible conversion of glycine and serine. In eukaryotes, the cytosolic and mitochondrial SHMT isoenzymes are encoded by distinct nuclear genes [37]. In yeast, Kastanos et al. [38] reported that the roles of the SHMT isoenzymes change as the nutritional requirements of the cell change, which will determine the direction of the reversible reaction. Specifically, cytosolic SHMT is the predominant contributor of glycine via the breakdown of serine when serine is sufficient, while mitochondrial SHMT functions preferentially in the direction of serine synthesis under conditions where serine is limiting. Serine accounts for quite a high ratio in amino acid composition of biomass protein and glucoamylase (Additional file 1) so it might be limiting in the fermentation. In this study, although the gene *An05g00410* encoding mitochondrial SHMT was overexpressed, mitochondrial SHMT in mutant OE-gly might not be a major contributor in converting serine into glycine and might function in the opposite direction, thus failing to elevate the intracellular glycine supply for glucoamylase overproduction. Similarly, the speculation proposed to account for the glucoamylase production performance of mutant OE-ala in shake flask fermentation might be that alanine transaminase encoded by the overexpressed gene *An11g02620* is not the major provider of alanine. Garcia-Campusano et al. [39] reported that in *S. cerevisiae*, alanine transaminase plays a catabolic role and the biosynthesis of alanine is achieved through an alternative pathway in aerobic glucose-grown culture. To sum up, the two mutants whose biosynthetic pathways have been engineered did not perform as what we designed, which is possibly due to the uncertainty of amino acid metabolism under different culture conditions.

In this study, we constructed five mutants to overexpress the genes involved in the limited amino acid synthetic pathways in the strain *A. niger* CBS 513.88 to improve the biosynthesis of these amino acids and the results are summarized in Fig. 5. The common strategies to improve the pool of a metabolite is upregulating the pathway enzymes to augment biosynthetic pathways and precursors or downregulating the competing pathways to decrease the synthesis of by-products [40] and we used the former one by introducing additional copies of the genes involved in the amino acid biosynthetic pathways. Results revealed that only mutant OE-asp2 out of the five mutants substantially improved the glucoamylase production. The engineering of amino acid biosynthetic pathways may generate unexpected results due to the complex regulation of amino acids and may exert more burden on cellular metabolism. Although our results provide a new idea for improving glucoamylase production, there still existed some shortcomings. The improvement of a certain amino acid biosynthesis certainly will trigger amino acid biosynthesis regulation, which alters the amino acid metabolism as well as the other intracellular amino acid pools. This may yield undesired results and it is difficult for us to figure out the details. For the cultivation of mutant OE-asp2 in shake flasks, we additionally carried out an experiment. We added exogenous aspartate in the culture medium and found no significant glucoamylase production improvement compared to its performance in shake flask culture without aspartate addition. Therefore, we speculated that in mutant OE-asp2 aspartate no longer serves as the limited amino acid for glucoamylase production. Moreover, based on our research, more efforts can be made to decipher the mechanism of synthesis and utilization of intracellular amino acids for glucoamylase production in mutant OE-asp2 and other mutants, which will give us new insights into the biosynthesis of glucoamylase in *A. niger*.

## Conclusion

Based on the previous integrative multi-omics prediction of limited amino acids for glucoamylase production in *A. niger* under oxygen-limited conditions, we constructed five mutants to strengthen the biosynthesis of these limited amino acids and then tested the glucoamylase productivity in shake flask cultivation. Fed-batch fermentation in the 5 L fermentor revealed that the glucoamylase activity of mutant OE-asp2 increased 60.3% than that of the parental strain *A. niger* CBS 513.88. Moreover, profiles of intracellular amino acid pools demonstrated that the reinforcement of cytosolic aspartate biosynthesis in mutant OE-asp2 elevated most intracellular amino acid pools, especially the pools of alanine, glycine and leucine. Therefore, we speculated that the glucoamylase production was significantly increased in mutant OE-asp2 mainly due to the increased supply of intracellular aspartate and other limiting amino acid precursors (alanine and glycine) derived from the improvement of cytosolic aspartate biosynthesis. Finally, our work demonstrated that aspartate plays an important role in the biosynthesis of amino acid precursors for glucoamylase production in *A. niger* under oxygen-limited conditions. Regulation of amino acid biosynthesis can efficiently relieve amino acid limitation in enzyme production. However, the amino acid ratio in biomass and protein products, together with the influence of amino acid biosynthetic pathways on intermediates and cofactors in global metabolism should be taken into consideration when selecting the target amino acids for engineering.

## Supplementary information

**Additional file 1:** Amino acid contents of biomass protein and glucoamylase.

**Additional file 2: Fig. S1**. Color changes of spores of *A. niger*. a. The parental strain CBS 513.88 b. Gene *olvA* defective mutant. **Fig. S2**. Schematic diagram of constructions of donor DNA for *olvA* deletion and target gene overexpression. **Fig. S3**. Schematic diagram of the generation of the mutants for gene overexpression by CRISPR/Cas9 assisted homologous recombination. **Fig. S4**. Comparison of the mutant CBS-Δ*olvA* and the parental strain CBS 513.88 in shake flask fermentation. Data represent the average values and standard deviations from three replicates. **Fig. S5**. Growth (a) and glucoamylase production (b) profiles of the two mutants OE-asp1 and OE-asp2 and the parental strain CBS 513.88 in the 5 L fermentor. The vertical line (at 40 h) shows the beginning of the oxygen-limited phase. Data represent the average values and standard deviations from three replicates. **Fig. S6**. a. Relative mRNA levels of gene *An04g06380* in the mutant OE-asp1. b. Relative mRNA levels of gene *An16g05570* in the mutant OE-asp2. Samples were taken at 36 h (exponential growth phase) and 72 h (oxygen-limited phase) from the 5 L fermentor. Data represent the average values and standard deviations from three replicates.

**Additional file 3: Table S1.** Primers for constructions of donor DNA for *olvA* deletion and target gene overexpression. **Table S2.** Primers for PCR diagnosis of transformants. **Table S3.** Primers for real-time quantitative PCR. **Table S4.** Plasmids used in the study

## Data Availability

All data generated or analysed during this study are included in this article and its supplementary information files.

## References

[1] Norouzian D, Akbarzadeh A, Scharer JM, Young MM (2006). Fungal glucoamylases. Biotechnol Adv.

[2] Driouch H, Sommer B, Wittmann C (2010). Morphology engineering of *Aspergillus niger* for improved enzyme production. Biotechnol Bioeng.

[3] Withers JM, Swift RJ, Wiebe MG, Robson GD, Punt PJ, van den Hondel CAMJJ, Trinci APJ (1998). Optimization and stability of glucoamylase production by recombinant strains of *Aspergillus niger* in chemostat culture. Biotechnol Bioeng.

[4] Bertolin ET, Costa JAV, Pasquali GDL (2001). Glucoamylase production in batch and fed-batch solid state fermentation: effect of maltose or starch addition. J Microbiol Biotechnol.

[5] Bertolin TE, Schmidell W, Maiorano AE, Casara J, Costa JAV (2003). Influence of carbon, nitrogen and phosphorous sources on glucoamylase production by *Aspergillus awamori* in solid state fermentation. Zeitschrift Fur Naturforschung C-a J Biosci.

[6] Pedersen L, Hansen K, Nielsen J, Lantz AE, Thykaer J (2012). Industrial glucoamylase fed-batch benefits from oxygen limitation and high osmolarity. Biotechnol Bioeng.

[7] Lu H, Cao W, Liu X, Sui Y, Ouyang L, Xia J, Huang M, Zhuang Y, Zhang S, Noorman H, Chu J (2018). Multi-omics integrative analysis with genome-scale metabolic model simulation reveals global cellular adaptation of *Aspergillus niger* under industrial enzyme production condition. Sci Rep.

[8] Gorgens JF, van Zyl WH, Knoetze JH, Hahn-Hagerdal B (2005). Amino acid supplementation improves heterologous protein production by *Saccharomyces cerevisiae* in defined medium. Appl Microbiol Biotechnol.

[9] Gorgens JF, Passoth V, van Zyl WH, Knoetze JH, Hahn-Hagerdal M (2005). Amino acid supplementation, controlled oxygen limitation and sequential double induction improves heterologous xylanase production by *Pichia stipitis*. FEMS Yeast Res.

[10] Heyland J, Fu JA, Blank LM, Schmid A (2011). Carbon metabolism limits recombinant protein production in *Pichia pastoris*. Biotechnol Bioeng.

[11] Xia XX, Qian ZG, Ki CS, Park YH, Kaplan DL, Lee SY (2010). Native-sized recombinant spider silk protein produced in metabolically engineered *Escherichia coli* results in a strong fiber. Proc Natl Acad Sci USA.

[12] Cao H, Parveen S, Ding D, Xu HJ, Tan TW, Liu L (2017). Metabolic engineering for recombinant major ampullate spidroin 2 (MaSp2) synthesis in *Escherichia coli*. Sci Rep.

[13] Zahrl RJ, Pena DA, Mattanovich D, Gasser B (2017). Systems biotechnology for protein production in *Pichia pastoris*. FEMS Yeast Res.

[14] Niederberger P, Miozzari G, Hütter R (1981). Biological role of the general control of amino acid biosynthesis in *Saccharomyces cerevisiae*. Mol Cell Biol.

[15] Carsiotis M, Jones RF (1974). Cross-pathway regulation: tryptophan-mediated control of histidine and arginine biosynthetic enzymes in *Neurospora crassa*. J Bacteriol.

[16] Carsiotis M, Jones RF, Wesseling AC (1974). Cross-pathway regulation: histidine-mediated control of histidine, tryptophan, and arginine biosynthetic enzymes in *Neurospora crassa*. J Bacteriol.

[17] Carsiotis M, Lacy AM (1965). Increased activity of tryptophan biosynthetic enzymes in histidine mutants of *Neurospora crassa*. J Bacteriol.

[18] Delforge J, Messenguy F, Wiame JM (1975). The regulation of arginine biosynthesis in *Saccharomyces cerevisiae.* The specificity of argR- mutations and the general control of amino-acid biosynthesis. Eur J Biochem.

[19] Messenguy F, Colin D (1980). HAVE J-PT: regulation of compartmentation of amino acid pools in *Saccharomyces cerevisiae* and its effects on metabolic control. Eur J Biochem.

[20] Verdoes JC, Punt PJ, Schrickx JM, van Verseveld HW, Stouthamer AH, van den Hondel CAMJJ (1993). Glucoamylase overexpression in *Aspergillus niger*: molecular genetic analysis of strains containing multiple copies of the*glaA* gene. Transgenic Res.

[21] An X, Ding C, Zhang H, Liu T, Li J (2019). Overexpression of *amyA* and *glaA* substantially increases glucoamylase activity in *Aspergillus niger*. Acta Biochim Biophys Sin.

[22] Krijgsheld P, Nitsche BM, Post H, Levin AM, Muller WH, Heck AJ, Ram AF, Altelaar AF, Wosten HA (2013). Deletion of *flbA* results in increased secretome complexity and reduced secretion heterogeneity in colonies of *Aspergillus niger*. J Proteome Res.

[23] Fiedler MRM, Barthel L, Kubisch C, Nai C, Meyer V (2018). Construction of an improved *Aspergillus niger* platform for enhanced glucoamylase secretion. Microbial Cell Factories.

[24] Lu H, Cao W, Ouyang L, Xia J, Huang M, Chu J, Zhuang Y, Zhang S, Noorman H (2017). Comprehensive reconstruction and in silico analysis of *Aspergillus niger* genome-scale metabolic network model that accounts for 1210 ORFs. Biotechnol Bioeng.

[25] Meyer V, Ram A, Punt P, Baltz R, Demain A, Davies J, Bull A, Junker B, Katz L, Lynd L, Masurekar P, Reeves C, Zhao H (2010). Genetics, genetic manipulation, and approaches to strain improvement of filamentous fungi. Manual of industrial microbiology and biotechnology.

[26] Jorgensen TR, Park J, Arentshorst M, van Welzen AM, Lamers G, Vankuyk PA, Damveld RA, van den Hondel CA, Nielsen KF, Frisvad JC, Ram AF (2011). The molecular and genetic basis of conidial pigmentation in *Aspergillus niger*. Fungal Genet Biol.

[27] Fujii I, Yasuoka Y, Tsai HF, Chang YC, Kwon-Chung KJ, Ebizuka Y (2004). Hydrolytic polyketide shortening by Ayg1p, a novel enzyme involved in fungal melanin biosynthesis. J Biol Chem.

[28] Wang G, Chu J, Zhuang YP, van Gulik W, Noorman H (2019). A dynamic model-based preparation of uniformly-C-13-labeled internal standards facilitates quantitative metabolomics analysis of *Penicillium chrysogenum*. J Biotechnol.

[29] Jouhten P, Rintala E, Huuskonen A, Tamminen A, Toivari M, Wiebe M, Ruohonen L, Penttila M, Maaheimo H (2008). Oxygen dependence of metabolic fluxes and energy generation of *Saccharomyces cerevisiae* CENPK113-1A. Bmc Syst Biol.

[30] Baumann K, Carnicer M, Dragosits M, Graf AB, Stadlmann J, Jouhten P, Maaheimo H, Gasser B, Albiol J, Mattanovich D, Ferrer P (2010). A multi-level study of recombinant *Pichia pastoris* in different oxygen conditions. Bmc Syst Biol.

[31] Hinnebusch AG (1986). The general control of amino acid biosynthetic genes in the yeast *Saccharomyces cerevisiae*. CRC.

[32] Braus GH (1991). Aromatic amino acid biosynthesis in the yeast *Saccharomyces cerevisiae*: a model system for the regulation of a eukaryotic biosynthetic pathway. Microbiol Rev.

[33] Hinnebusch AG (1988). Mechanisms of gene regulation in the general control of amino acid biosynthesis in *Saccharomyces cerevisiae*. Microbiol Rev.

[34] Easlon E, Tsang F, Skinner C, Wang C, Lin SJ (2008). The malate-aspartate NADH shuttle components are novel metabolic longevity regulators required for calorie restriction-mediated life span extension in yeast. Genes Dev.

[35] Cao JX, Barbosa JM, Singh NK, Locy RD (2013). GABA shunt mediates thermotolerance in *Saccharomyces cerevisiae* by reducing reactive oxygen production. Yeast.

[36] Kumar S, Punekar NS (1997). The metabolism of 4-aminobutyrate (GABA) in fungi. Mycol Res.

[37] Appling DR (1991). Compartmentation of folate-mediated one-carbon metabolism in eukaryotes. FASEB J.

[38] Kastanos EK, Woldman YY, Appling DR (1997). Role of mitochondrial and cytoplasmic serine hydroxymethyltransferase isozymes in de novo purine synthesis in *Saccharomyces cerevisiae*. Biochemistry.

[39] Garcia-Campusano F, Anaya VH, Robledo-Arratia L, Quezada H, Hernandez H, Riego L, Gonzalez A (2009). ALT1-encoded alanine aminotransferase plays a central role in the metabolism of alanine in *Saccharomyces cerevisiae*. Can J Microbiol.

[40] Liu Q, Yu T, Campbell K, Nielsen J, Chen Y (2018). Modular pathway rewiring of yeast for amino acid production. Methods Enzymol.

