# Supplementary material for: Improving cytosolic aspartate biosynthesis increases glucoamylase production in Aspergillus niger under oxygen limitation
Source: Microb Cell Fact. 2020 Apr 3;19:81. doi: 10.1186/s12934-020-01340-1 (PMC7118866; doi:10.1186/s12934-020-01340-1)
Supplement: Supplementary file 1 — Additional file 1: Amino acid contents of biomass protein and glucoamylase. [file 12934_2020_1340_MOESM1_ESM.docx]

Additional file 1: Amino acid contents of biomass protein and glucoamylase

| **Amino acids** | **Contents of glucoamylase (%)** | **Contents of biomass protein (%)** |
| --- | --- | --- |
| Alanine | 10.16 | 11.2 |
| Cysteine | 1.56 | 0.86 |
| Aspartate | 6.88 | 6.80 |
| Glutamate | 4.06 | 9.73 |
| Phenylalanine | 3.44 | 3.38 |
| Glycine | 7.34 | 9.33 |
| Histidine | 0.63 | 2.22 |
| Isoleucine | 3.75 | 4.29 |
| Lysine | 2.03 | 7.05 |
| Leucine | 7.50 | 7.62 |
| Methionine | 0.47 | 1.14 |
| Asparagine | 3.91 | 2.26 |
| Proline | 3.44 | 4.78 |
| Glutamine | 2.66 | 3.25 |
| Arginine | 3.13 | 4.47 |
| Serine | 13.75 | 6.80 |
| Threonine | 11.56 | 5.45 |
| Valine | 6.56 | 5.80 |
| Tryptophan | 2.97 | 1.36 |
| Tyrosine | 4.22 | 2.22 |
