# Supplementary material for: Improving cytosolic aspartate biosynthesis increases glucoamylase production in Aspergillus niger under oxygen limitation
Source: Microb Cell Fact. 2020 Apr 3;19:81. doi: 10.1186/s12934-020-01340-1 (PMC7118866; doi:10.1186/s12934-020-01340-1)
Supplement: Supplementary file 2 — Additional file 2: Fig. S1. Color changes of spores of A. niger. a. The parental strain CBS 513.88 b. Gene olvA defective mutant. Fig. S2. Schematic diagram of constructions of donor DNA for olvA deletion and target gene overexpression. Fig. S3. Schematic diagram of the generation of the mutants for gene overexpression by CRISPR/Cas9 assisted homologous recombination. Fig. S4. Comparison of the mutant CBS-ΔolvA and the parental strain CBS 513.88 in shake flask fermentation. Data represent the average values and standard deviations from three replicates. Fig. S5. Growth (a) and glucoamylase production (b) profiles of the two mutants OE-asp1 and OE-asp2 and the parental strain CBS 513.88 in the 5 L fermentor. The vertical line (at 40 h) shows the beginning of the oxygen-limited phase. Data represent the average values and standard deviations from three replicates. Fig. S6. a. Relative mRNA levels of gene An04g06380 in the mutant OE-asp1. b. Relative mRNA levels of gene An16g05570 in the mutant OE-asp2. Samples were taken at 36 h (exponential growth phase) and 72 h (oxygen-limited phase) from the 5 L fermentor. Data represent the average values and standard deviations from three replicates. [file 12934_2020_1340_MOESM2_ESM.docx]

**List of supplementary figures**

Fig. S1. Color changes of spores of *A. niger*. a. The parental strain CBS 513.88 b. Gene *olvA* defective mutant.

Fig. S2. Schematic diagram of constructions of donor DNA for *olvA* deletion and target gene overexpression.

Fig. S3. Schematic diagram of the generation of the mutants for gene overexpression by CRISPR/Cas9 assisted homologous recombination.

Fig. S4. Comparison of the mutant CBS-Δ*olvA* and the parental strain CBS 513.88 in shake flask fermentation. Data represent the average values and standard deviations from three replicates.

Fig. S5. Growth (a) and glucoamylase production (b) profiles of the two mutants OE-asp1 and OE-asp2 and the parental strain CBS 513.88 in the 5L fermentor. The vertical line (at 40 h) shows the beginning of the oxygen-limited phase. Data represent the average values and standard deviations from three replicates.

Fig. S6. a. Relative mRNA levels of gene *An04g06380* in the mutant OE-asp1. b. Relative mRNA levels of gene *An16g05570* in the mutant OE-asp2. Samples were taken at 36h (exponential growth phase) and 72h (oxygen-limited phase) from the 5L fermentor. Data represent the average values and standard deviations from three replicates.

**
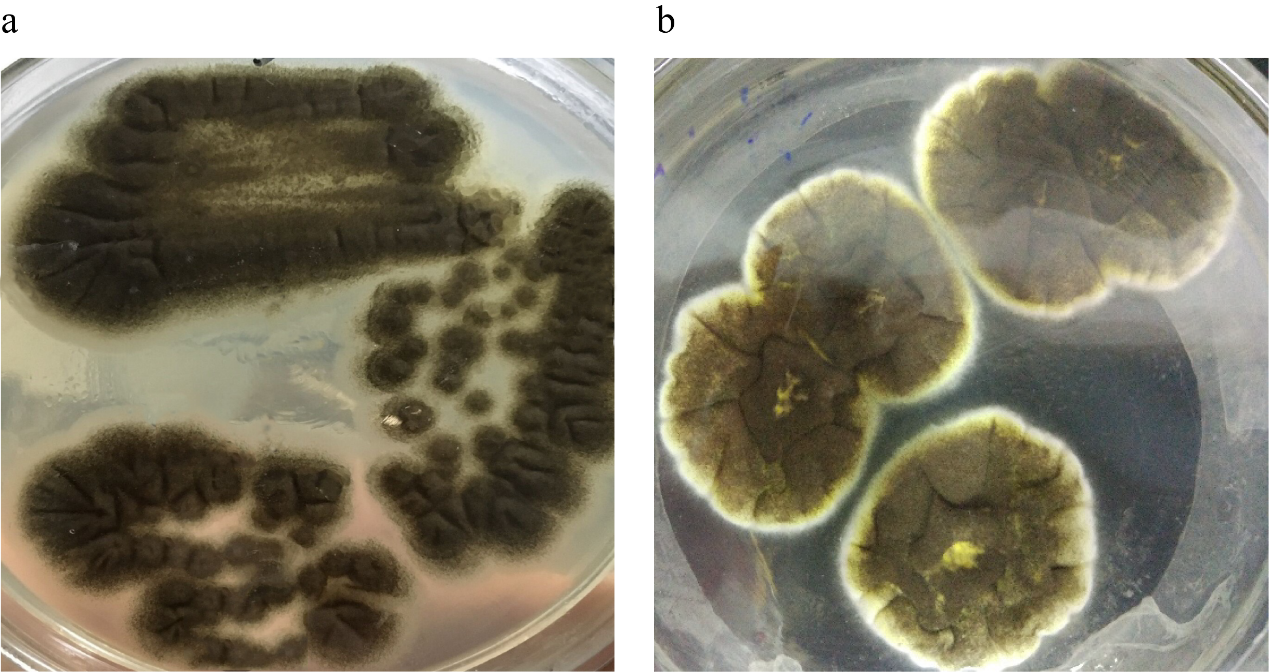
**

Fig. S1. Color changes of spores of *A. niger*. a. The parental strain CBS 513.88 b. Gene *olvA* defective mutant.





Fig. S2. Schematic diagram of constructions of donor DNA for *olvA* deletion and target gene overexpression.





Fig. S3. Schematic diagram of the generation of the mutants for gene overexpression by CRISPR/Cas9 assisted homologous recombination.


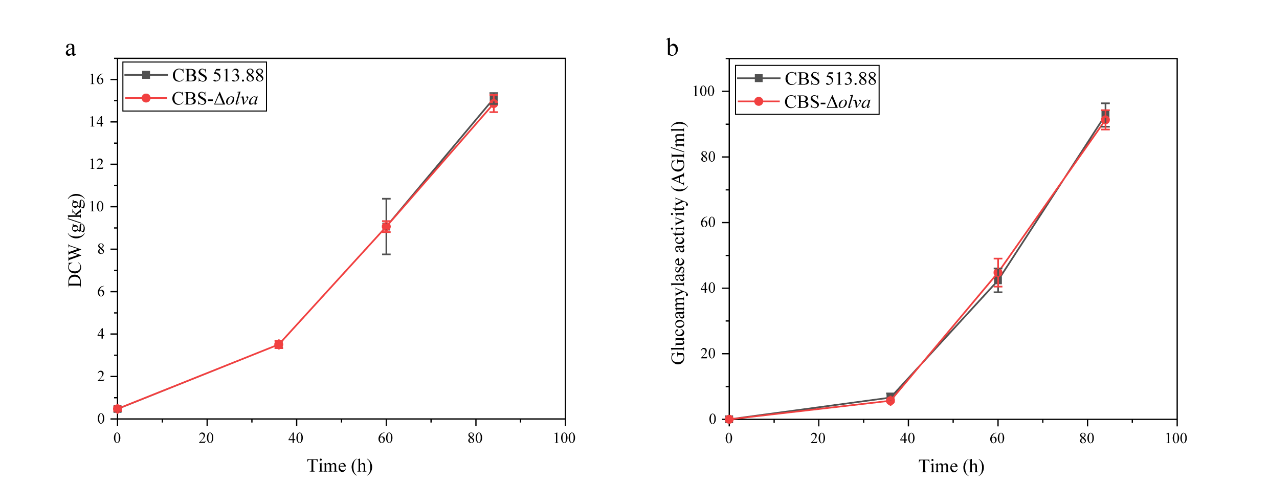


Fig. S4. Comparison of the mutant CBS-Δ*olvA* and the parental strain CBS 513.88 in shake flask fermentation. Data represent the average values and standard deviations from three replicates.


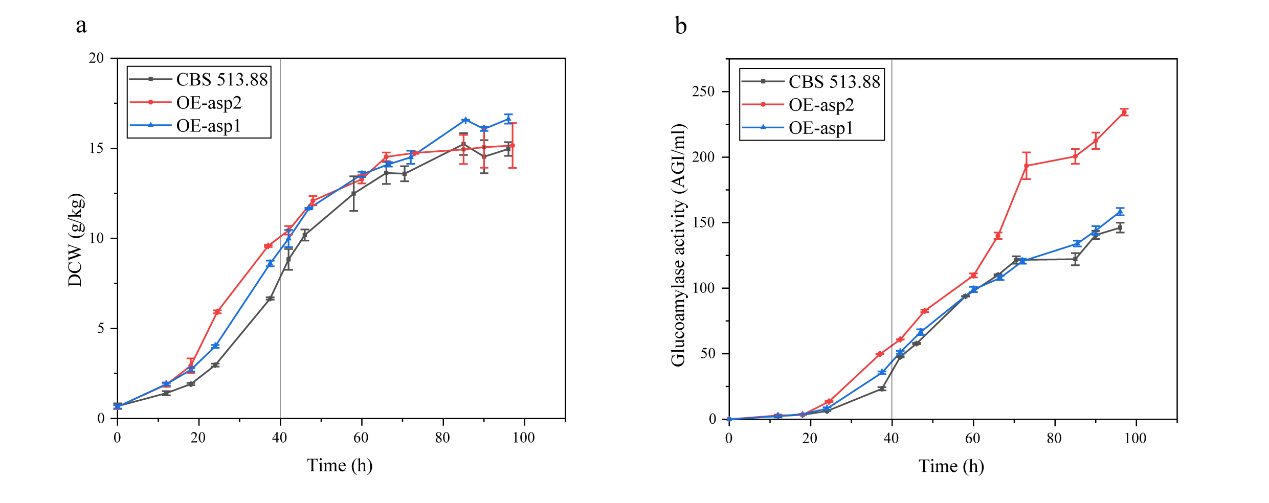


Fig. S5. Growth (a) and glucoamylase production (b) profiles of the two mutants OE-asp1 and OE-asp2 and the parental strain CBS 513.88 in the 5L fermentor. The vertical line (at 40 h) shows the beginning of the oxygen-limited phase. Data represent the average values and standard deviations from three replicates.


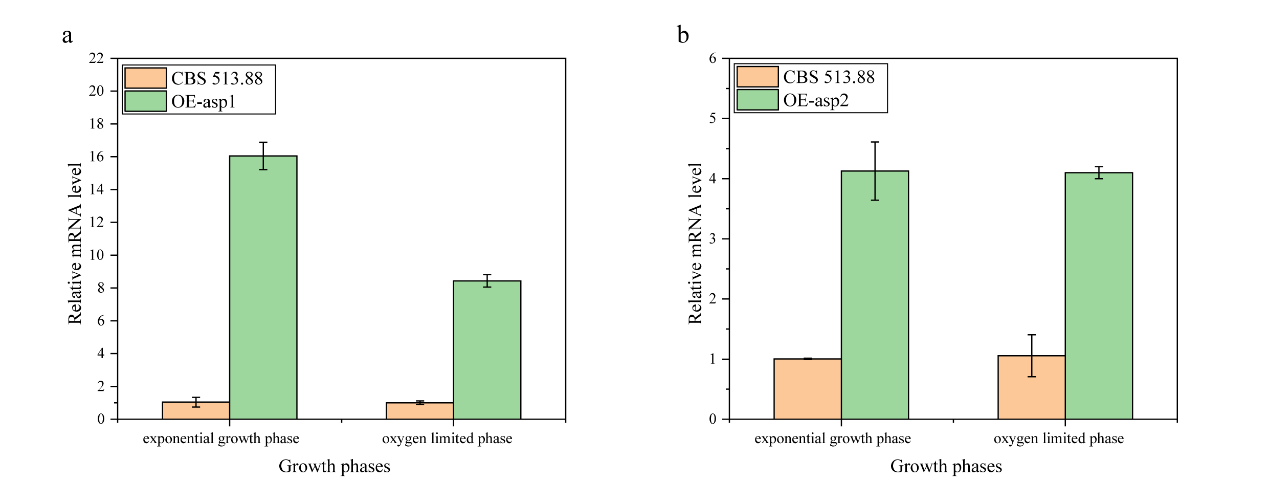


Fig. S6. a. Relative mRNA levels of gene *An04g06380* in the mutant OE-asp1. b. Relative mRNA levels of gene *An16g05570* in the mutant OE-asp2. Samples were taken at 36h (exponential growth phase) and 72h (oxygen-limited phase) from the 5L fermentor. Data represent the average values and standard deviations from three replicates.
