# Supplementary material for: Improving cytosolic aspartate biosynthesis increases glucoamylase production in Aspergillus niger under oxygen limitation
Source: Microb Cell Fact. 2020 Apr 3;19:81. doi: 10.1186/s12934-020-01340-1 (PMC7118866; doi:10.1186/s12934-020-01340-1)
Supplement: Supplementary file 3 — Additional file 3: Table S1. Primers for constructions of donor DNA for olvA deletion and target gene overexpression. Table S2. Primers for PCR diagnosis of transformants. Table S3. Primers for real-time quantitative PCR. Table S4. Plasmids used in the study [file 12934_2020_1340_MOESM3_ESM.docx]

**List of supplementary tables**

Table S1 Primers for constructions of donor DNA for *olvA* deletion and target gene overexpression

Table S2 Primers for PCR diagnosis of transformants

Table S3 Primers for real-time quantitative PCR

Table S4 Plasmids used in the study

Table S1 Primers for constructions of donor DNA for *olvA* deletion and target gene overexpression

| Primers | Sequences (5’ to 3’) |
| --- | --- |
| Primers of PCR amplification of expression cassette (P*gpdA*+*hygR* or candidate gene+T*trpC*) | |
| PXT-F | agcaaacacaGAATTCCCTTGTATCTCTACACAC |
| PXT-R | accatcctttTCGAGTGGAGATGTGGAGTG |
| Primers of PCR amplification of upstream and downstream homologous arm of olvA | |
| *olvA*-up-1 | TCTGCACGCTAGCCCTTGATG |
| *olvA*-up-2 | aagggaattcTGTGTTTGCTTGCTTGTTCGC |
| *olvA*-dn-1 | ctccactcgaAAAGGATGGTTATTATCGGTG |
| *olvA*-dn-2 | CAAAGTACTACACCACCAGAC |
| Primers of PCR amplification of pAN 7-1 excluding the hygromycin gene | |
| *ΔhygR*-F | AGTAGATGCCGACCGCGGGAT |
| *ΔhygR*-R | GGTGATGTCTGCTCAAGCGG |
| Primers of PCR amplification of the candidate genes from the genome of CBS 513.88 | |
| *An11g02620*-F | ctaccccgcttgagcagacatcaccATGACCTGGCAAATTAAGCTCGCCT |
| *An11g02620*-R | gtggatcccgcggtcggcatctactTTACTTGTACTTGGCCATGAACTCC |
| *An04g00990*-F | ctaccccgcttgagcagacatcaccATGTCTAACCTTCCTCACGAGCCCG |
| *An04g00990*-R | gtggatcccgcggtcggcatctactTTACCACCAGTCACCCTGGTCCTTC |
| *An05g00410*-F | ctaccccgcttgagcagacatcaccATGTTGAGTCGCTGTGGACGTCAGG |
| *An05g00410*-R | gtggatcccgcggtcggcatctactCTACTCATCCTTAGCCCAGGGCAGA |
| *An04g06380*-F | ctaccccgcttgagcagacatcaccATGCTGTCCACCCTCAGAGTCG |
| *An04g06380*-R | gtggatcccgcggtcggcatctactTTAACCGGTGATCTTGTAGATGG |
| *An16g05570*-F | ctaccccgcttgagcagacatcaccATGTCGCCCCTTTCCTCCTCTTCTT |
| *An16g05570*-R | gtggatcccgcggtcggcatctactTTATGAAGTCTCCCGAACTACGCTG |

The lowercase lettes of the sequences represent overlapping sequences

Table S2 Primers for PCR diagnosis of transformants

| Primers | Sequences (5’ to 3’) |
| --- | --- |
| P1 | TGCAGCAACACATACTCCAAGAAG |
| P2 | GTTTGAAACCATGTCTGAGCGTAG |
| P3 | CGTCCTCCGACTTCAATAAGGAGC |
| P5 | GAGGCACTCGAAAATAAGGCGTCAGC |
| P6 | GCTTCTTGAAATCCTCCGGGTTGGAG |

Table S3 Primers for real-time quantitative PCR

| Primers | Sequences (5’ to 3’) |
| --- | --- |
| *An11g02620*-rt-F | AGCGCATGGAGGGTGTTGAATG |
| *An11g02620*-rt-R | TCAAGGAGACGGAGGCAGTAG |
| *An04g00990*-rt-F | GTCTAACCTTCCTCACGAGC |
| *An04g00990*-rt-R | ACACGGAACTGGATGACAC |
| *An05g00410*-rt-F | CGCCATCTATGACATTCTCCAG |
| *An05g00410*-rt-R | GCTTGCGATGTGAAGTTCTC |
| *An04g06380*-rt-F | CAGAGTTTCGCCAAGAACATG |
| *An04g06380*-rt-R | TGGGAGGGTTGGAGTAGAAG |
| *An16g05570*-rt-F | GGTCGATCTAGTCATTGGCG |
| *An16g05570*-rt-R | GGGAGGTATTCGTGATTGAGG |
| *GAPDH*-rt-F | CTCCCAAGGTCGGTATCAAC |
| *GAPDH*-rt-R | GCATAGTGGGTCTCGATGAAG |

Table S4 Plasmids used in the study

| Plasmids | Sequences (5’ to 3’) |
| --- | --- |
| pAN 7-1 | GAATTCCCTTGTATCTCTACACACAGGCTCAAATCAATAAGAAGAACGGTTCGTCTTTTTCGTTTATATCTTGCATCGTCCCAAAGCTATTGGCGGGATATTCTGTTTGCAGTTGGCTGACTTGAAGTAATCTCTGCAGATCTTTCGACACTGAAATACGTCGAGCCTGCTCCGCTTGGAAGCGGCGAGGAGCCTCGTCCTGTCACAACTACCAACATGGAGTACGATAAGGGCCAGTTCCGCCAGCTCATTAAGAGCCAGTTCATGGGCGTTGGCATGATGGCCGTCATGCATCTGTACTTCAAGTACACCAACGCTCTTCTGATCCAGTCGATCATCCGCTGAAGGCGCTTTCGAATCTGGTTAAGATCCACGTCTTCGGGAAGCCAGCGACTGGTGACCTCCAGCGTCCCTTTAAGGCTGCCAACAGCTTTCTCAGCCAGGGCCAGCCCAAGACCGACAAGGCCTCCCTCCAGAACGCCGAGAAGAACTGGAGGGGTGGTGTCAAGGAGGAGTAAGCTCCTTATTGAAGTCGGAGGACGGAGCGGTGTCAAGAGGATATTCTTCGACTCTGTATTATAGATAAGATGATGAGGAATTGGAGGTAGCATAGCTTCATTTGGATTTGCTTTCCAGGCTGAGACTCTAGCTTGGAGCATAGAGGGTCCTTTGGCTTTCAATATTCTCAAGTATCTCGAGTTTGAACTTATTCCCTGTGAACCTTTTATTCACCAATGAGCATTGGAATGAACATGAATCTGAGGACTGCAATCGCCATGAGGTTTTCGAAATACATCCGGATGTCGAAGGCTTGGGGCACCTGCGTTGGTTGAATTTAGAACGTGGCACTATTGATCATCCGATAGCTCTGCAAAGGGCGTTGCACAATGCAAGTCAAACGTTGCTAGCAGTTCCAGGTGGAATGTTATGATGAGCATTGTATTAAATCAGGAGATATAGCATGATCTCTAGTTAGCTCACCACAAAAGTCAGACGGCGTAACCAAAAGTCACACAACACAAGCTGTAAGGATTTCGGCACGGCTACGGAAGACGGAGAAGCCACCTTCAGTGGACTCGAGTACCATTTAATTCTATTTGTGTTTGATCGAGACCTAATACAGCCCCTACAACGACCATCAAAGTCGTATAGCTACCAGTGAGGAAGTGGACTCAAATCGACTTCAGCAACATCTCCTGGATAAACTTTAAGCCTAAACTATACAGAATAAGATAGGTGGAGAGCTTATACCGAGCTCCCAAATCTGTCCAGATCATGGTTGACCGGTGCCTGGATCTTCCTATAGAATCATCCTTATTCGTTGACCTAGCTGATTCTGGAGTGACCCAGAGGGTCATGACTTGAGCCTAAAATCCGCCGCCTCCACCATTTGTAGAAAAATGTGACGAACTCGTGAGCTCTGTACAGTGACCGGTGACTCTTTCTGGCATGCGGAGAGACGGACGGACGCAGAGAGAAGGGCTGAGTAATAAGCCACTGGCCAGACAGCTCTGGCGGCTCTGAGGTGCAGTGGATGATTATTAATCCGGGACCGGCCGCCCCTCCGCCCCGAAGTGGAAAGGCTGGTGTGCCCCTCGTTGACCAAGAATCTATTGCATCATCGGAGAATATGGAGCTTCATCGAATCACCGGCAGTAAGCGAAGGAGAATGTGAAGCCAGGGGTGTATAGCCGTCGGCGAAATAGCATGCCATTAACCTAGGTACAGAAGTCCAATTGCTTCCGATCTGGTAAAAGATTCACGAGATAGTACCTTCTCCGAAGTAGGTAGAGCGAGTACCCGGCGCGTAAGCTCCCTAATTGGCCCATCCGGCATCTGTAGGGCGTCCAAATATCGTGCCTCTCCTGCTTTGCCCGGTGTATGAAACCGGAAAGGCCGCTCAGGAGCTGGCCAGCGGCGCAGACCGGGAACACAAGCTGGCAGTCGACCCATCCGGTGCTCTGCACTCGACCTGCTGAGGTCCCTCAGTCCCTGGTAGGCAGCTTTGCCCCGTCTGTCCGCCCGGTGTGTCGGCGGGGTTGACAAGGTCGTTGCGTCAGTCCAACATTTGTTGCCATATTTTCCTGCTCTCCCCACCAGCTGCTCTTTTCTTTTCTCTTTCTTTTCCCATCTTCAGTATATTCATCTTCCCATCCAAGAACCTTTATTTCCCCTAAGTAAGTACTTTGCTACATCCATACTCCATCCTTCCCATCCCTTATTCCTTTGAACCTTTCAGTTCGAGCTTTCCCACTTCATCGCAGCTTGACTAACAGCTACCCCGCTTGAGCAGACATCACCATGCCTGAACTCACCGCGACGTCTGTCGAGAAGTTTCTGATCGAAAAGTTCGACAGCGTCTCCGACCTGATGCAGCTCTCGGAGGGCGAAGAATCTCGTGCTTTCAGCTTCGATGTAGGAGGGCGTGGATATGTCCTGCGGGTAAATAGCTGCGCCGATGGTTTCTACAAAGATCGTTATGTTTATCGGCACTTTGCATCGGCCGCGCTCCCGATTCCGGAAGTGCTTGACATTGGGGAATTCAGCGAGAGCCTGACCTATTGCATCTCCCGCCGTGCACAGGGTGTCACGTTGCAAGACCTGCCTGAAACCGAACTGCCCGCTGTTCTGCAGCCGGTCGCGGAGGCCATGGATGCGATCGCTGCGGCCGATCTTAGCCAGACGAGCGGGTTCGGCCCATTCGGACCGCAAGGAATCGGTCAATACACTACATGGCGTGATTTCATATGCGCGATTGCTGATCCCCATGTGTATCACTGGCAAACTGTGATGGACGACACCGTCAGTGCGTCCGTCGCGCAGGCTCTCGATGAGCTGATGCTTTGGGCCGAGGACTGCCCCGAAGTCCGGCACCTCGTGCACGCGGATTTCGGCTCCAACAATGTCCTGACGGACAATGGCCGCATAACAGCGGTCATTGACTGGAGCGAGGCGATGTTCGGGGATTCCCAATACGAGGTCGCCAACATCTTCTTCTGGAGGCCGTGGTTGGCTTGTATGGAGCAGCAGACGCGCTACTTCGAGCGGAGGCATCCGGAGCTTGCAGGATCGCCGCGGCTCCGGGCGTATATGCTCCGCATTGGTCTTGACCAACTCTATCAGAGCTTGGTTGACGGCAATTTCGATGATGCAGCTTGGGCGCAGGGTCGATGCGACGCAATCGTCCGATCCGGAGCCGGGACTGTCGGGCGTACACAAATCGCCCGCAGAAGCGCGGCCGTCTGGACCGATGGCTGTGTAGAAGTACTCGCCGATAGTGGAAACCGACGCCCCAGCACTCGTCCGAGGGCAAAGGAATAGAGTAGATGCCGACCGCGGGATCCACTTAACGTTACTGAAATCATCAAACAGCTTGACGAATCTGGATATAAGATCGTTGGTGTCGATGTCAGCTCCGGAGTTGAGACAAATGGTGTTCAGGATCTCGATAAGATACGTTCATTTGTCCAAGCAGCAAAGAGTGCCTTCTAGTGATTTAATAGCTCCATGTCAACAAGAATAAAACGCGTTTTCGGGTTTACCTCTTCCAGATACAGCTCATCTGCAATGCATTAATGCATTGACTGCAACCTAGTAACGCCTTNCAGGCTCCGGCGAAGAGAAGAATAGCTTAGCAGAGCTATTTTCATTTTCGGGAGACGAGATCAAGCAGATCAACGGTCGTCAAGAGACCTACGAGACTGAGGAATCCGCTCTTGGCTCCACGCGACTATATATTTGTCTCTAATTGTACTTTGACATGCTCCTCTTCTTTACTCTGATAGCTTGACTATGAAAATTCCGTCACCAGCNCCTGGGTTCGCAAAGATAATTGCATGTTTCTTCCTTGAACTCTCAAGCCTACAGGACACACATTCATCGTAGGTATAAACCTCGAAATCANTTCCTACTAAGATGGTATACAATAGTAACCATGCATGGTTGCCTAGTGAATGCTCCGTAACACCCAATACGCCGGCCGAAACTTTTTTACAACTCTCCTATGAGTCGTTTACCCAGAATGCACAGGTACACTTGTTTAGAGGTAATCCTTCTTTCTAGAAGTCCTCGTGTACTGTGTAAGCGCCCACTCCACATCTCCACTCGACCTGCAGGCATGCAAGCTTGGCACTGGCCGTCGTTTTACAACGTCGTGACTGGGAAAACCCTGGCGTTACCCAACTTAATCGCCTTGCAGCACATCCCCCTTTCGCCAGCTGGCGTAATAGCGAAGAGGCCCGCACCGATCGCCCTTCCCAACAGTTGCGCAGCCTGAATGGCGAATGGCGCCTGATGCGGTATTTTCTCCTTACGCATCTGTGCGGTATTTCACACCGCATATGGTGCACTCTCAGTACAATCTGCTCTGATGCCGCATAGTTAAGCCAGCCCCGACACCCGCCAACACCCGCTGACGCGCCCTGACGGGCTTGTCTGCTCCCGGCATCCGCTTACAGACAAGCTGTGACCGTCTCCGGGAGCTGCATGTGTCAGAGGTTTTCACCGTCATCACCGAAACGCGCGAGACGAAAGGGCCTCGTGATACGCCTATTTTTATAGGTTAATGTCATGATAATAATGGTTTCTTAGACGTCAGGTGGCACTTTTCGGGGAAATGTGCGCGGAACCCCTATTTGTTTATTTTTCTAAATACATTCAAATATGTATCCGCTCATGAGACAATAACCCTGATAAATGCTTCAATAATATTGAAAAAGGAAGAGTATGAGTATTCAACATTTCCGTGTCGCCCTTATTCCCTTTTTTGCGGCATTTTGCCTTCCTGTTTTTGCTCACCCAGAAACGCTGGTGAAAGTAAAAGATGCTGAAGATCAGTTGGGTGCACGAGTGGGTTACATCGAACTGGATCTCAACAGCGGTAAGATCCTTGAGAGTTTTCGCCCCGAAGAACGTTTTCCAATGATGAGCACTTTTAAAGTTCTGCTATGTGGCGCGGTATTATCCCGTATTGACGCCGGGCAAGAGCAACTCGGTCGCCGCATACACTATTCTCAGAATGACTTGGTTGAGTACTCACCAGTCACAGAAAAGCATCTTACGGATGGCATGACAGTAAGAGAATTATGCAGTGCTGCCATAACCATGAGTGATAACACTGCGGCCAACTTACTTCTGACAACGATCGGAGGACCGAAGGAGCTAACCGCTTTTTTGCACAACATGGGGGATCATGTAACTCGCCTTGATCGTTGGGAACCGGAGCTGAATGAAGCCATACCAAACGACGAGCGTGACACCACGATGCCTGTAGCAATGGCAACAACGTTGCGCAAACTATTAACTGGCGAACTACTTACTCTAGCTTCCCGGCAACAATTAATAGACTGGATGGAGGCGGATAAAGTTGCAGGACCACTTCTGCGCTCGGCCCTTCCGGCTGGCTGGTTTATTGCTGATAAATCTGGAGCCGGTGAGCGTGGGTCTCGCGGTATCATTGCAGCACTGGGGCCAGATGGTAAGCCCTCCCGTATCGTAGTTATCTACACGACGGGGAGTCAGGCAACTATGGATGAACGAAATAGACAGATCGCTGAGATAGGTGCCTCACTGATTAAGCATTGGTAACTGTCAGACCAAGTTTACTCATATATACTTTAGATTGATTTAAAACTTCATTTTTAATTTAAAAGGATCTAGGTGAAGATCCTTTTTGATAATCTCATGACCAAAATCCCTTAACGTGAGTTTTCGTTCCACTGAGCGTCAGACCCCGTAGAAAAGATCAAAGGATCTTCTTGAGATCCTTTTTTTCTGCGCGTAATCTGCTGCTTGCAAACAAAAAAACCACCGCTACCAGCGGTGGTTTGTTTGCCGGATCAAGAGCTACCAACTCTTTTTCCGAAGGTAACTGGCTTCAGCAGAGCGCAGATACCAAATACTGTCCTTCTAGTGTAGCCGTAGTTAGGCCACCACTTCAAGAACTCTGTAGCACCGCCTACATACCTCGCTCTGCTAATCCTGTTACCAGTGGCTGCTGCCAGTGGCGATAAGTCGTGTCTTACCGGGTTGGACTCAAGACGATAGTTACCGGATAAGGCGCAGCGGTCGGGCTGAACGGGGGGTTCGTGCACACAGCCCAGCTTGGAGCGAACGACCTACACCGAACTGAGATACCTACAGCGTGAGCTATGAGAAAGCGCCACGCTTCCCGAAGGGAGAAAGGCGGACAGGTATCCGGTAAGCGGCAGGGTCGGAACAGGAGAGCGCACGAGGGAGCTTCCAGGGGGAAACGCCTGGTATCTTTATAGTCCTGTCGGGTTTCGCCACCTCTGACTTGAGCGTCGATTTTTGTGATGCTCGTCAGGGGGGCGGAGCCTATGGAAAAACGCCAGCAACGCGGCCTTTTTACGGTTCCTGGCCTTTTGCTGGCCTTTTGCTCACATGTTCTTTCCTGCGTTATCCCCTGATTCTGTGGATAACCGTATTACCGCCTTTGAGTGAGCTGATACCGCTCGCCGCAGCCGAACGACCGAGCGCAGCGAGTCAGTGAGCGAGGAAGCGGAAGAGCGCCCAATACGCAAACCGCCTCTCCCCGCGCGTTGGCCGATTCATTAATGCAGCTGGCACGACAGGTTTCCCGACTGGAAAGCGGGCAGTGAGCGCAACGCAATTAATGTGAGTTAGCTCACTCATTAGGCACCCCAGGCTTTACACTTTATGCTTCCGGCTCGTATGTTGTGTGGAATTGTGAGCGGATAACAATTTCACACAGGAAACAGCTATGACCATGATTAC |
| pAN-*olvA* | GAATTCCCTTGTATCTCTACACACAGGCTCAAATCAATAAGAAGAACGGTTCGTCTTTTTCGTTTATATCTTGCATCGTCCCAAAGCTATTGGCGGGATATTCTGTTTGCAGTTGGCTGACTTGAAGTAATCTCTGCAGATCTTTCGACACTGAAATACGTCGAGCCTGCTCCGCTTGGAAGCGGCGAGGAGCCTCGTCCTGTCACAACTACCAACATGGAGTACGATAAGGGCCAGTTCCGCCAGCTCATTAAGAGCCAGTTCATGGGCGTTGGCATGATGGCCGTCATGCATCTGTACTTCAAGTACACCAACGCTCTTCTGATCCAGTCGATCATCCGCTGAAGGCGCTTTCGAATCTGGTTAAGATCCACGTCTTCGGGAAGCCAGCGACTGGTGACCTCCAGCGTCCCTTTAAGGCTGCCAACAGCTTTCTCAGCCAGGGCCAGCCCAAGACCGACAAGGCCTCCCTCCAGAACGCCGAGAAGAACTGGAGGGGTGGTGTCAAGGAGGAGTAAGCTCCTTATTGAAGTCGGAGGACGGAGCGGTGTCAAGAGGATATTCTTCGACTCTGTATTATAGATAAGATGATGAGGAATTGGAGGTAGCATAGCTTCATTTGGATTTGCTTTCCAGGCTGAGACTCTAGCTTGGAGCATAGAGGGTCCTTTGGCTTTCAATATTCTCAAGTATCTCGAGTTTGAACTTATTCCCTGTGAACCTTTTATTCACCAATGAGCATTGGAATGAACATGAATCTGAGGACTGCAATCGCCATGAGGTTTTCGAAATACATCCGGATGTCGAAGGCTTGGGGCACCTGCGTTGGTTGAATTTAGAACGTGGCACTATTGATCATCCGATAGCTCTGCAAAGGGCGTTGCACAATGCAAGTCAAACGTTGCTAGCAGTTCCAGGTGGAATGTTATGATGAGCATTGTATTAAATCAGGAGATATAGCATGATCTCTAGTTAGCTCACCACAAAAGTCAGACGGCGTAACCAAAAGTCACACAACACAAGCTGTAAGGATTTCGGCACGGCTACGGAAGACGGAGAAGCCACCTTCAGTGGACTCGAGTACCATTTAATTCTATTTGTGTTTGATCGAGACCTAATACAGCCCCTACAACGACCATCAAAGTCGTATAGCTACCAGTGAGGAAGTGGACTCAAATCGACTTCAGCAACATCTCCTGGATAAACTTTAAGCCTAAACTATACAGAATAAGATAGGTGGAGAGCTTATACCGAGCTCCCAAATCTGTCCAGATCATGGTTGACCGGTGCCTGGATCTTCCTATAGAATCATCCTTATTCGTTGACCTAGCTGATTCTGGAGTGACCCAGAGGGTCATGACTTGAGCCTAAAATCCGCCGCCTCCACCATTTGTAGAAAAATGTGACGAACTCGTGAGCTCTGTACAGTGACCGGTGACCGGAGAGACGGACGGACGCAGAGAGAAGGGCTGAGTAATAAGCCACTGGCCAGACAGCTCTGGCGGCTCTGAGGTGCAGTGGATGATTATTAATCCGGGACCGGCCGCCCCTCCGCCCCGAAGTGGAAAGGCTGGTGTGCCCCTCGTTGACCAAGAATCTATTGCATCATCGGAGAATATGGAGCTTCATCGAATCACCGGCAGTAAGCGAAGGAGAATGTGAAGCCAGGGGTGTATAGCCGTCGGCGAAATAGCATGCCATTAACCTAGGTACAGAAGTCCAATTGCTTCCGATCTGGTAAAAGATTCACGAGATAGTACCTTCTCCGAAGTAGGTAGAGCGAGTACCCGGCGCGTAAGCTCCCTAATTGGCCCATCCGGCATCTGTAGGGCGTCCAAATATCGTGCCTCTCCTGCTTTGCCCGGTGTATGAAACCGGAAAGGCCGCTCAGGAGCTGGCCAGCGGCGCAGACCGGGAACACAAGCTGGCAGTCGACCCATCCGGTGCTCTGCACTCGACCTGCTGAGGTCCCTCAGTCCCTGGTAGGCAGCTTTGCCCCGTCTGTCCGCCCGGTGTGTCGGCGGGGTTGACAAGGTCGTTGCGTCAGTCCAACATTTGTTGCCATATTTTCCTGCTCTCCCCACCAGCTGCTCTTTTCTTTTCTCTTTCTTTTCCCATCTTCAGTATATTCATCTTCCCATCCAAGAACCTTTATTTCCCCTAAGTAAGTACTTTGCTACATCCATACTCCATCCTTCCCATCCCTTATTCCTTTGAACCTTTCAGTTCGAGCTTTCCCACTTCATCGCAGCTTGACTAACAGCTACCCCGCTTGAGCAGACATCACCATGCCTGAACTCACCGCGACGTGACTATAAGGACCACGACGGAGACTACAAGGATCATGATATTGATTACAAAGACGATGACGATAAGATGGCCCCAAAGAAGAAGCGGAAGGTCGGTATCCACGGAGTCCCAGCAGCCGACAAGAAGTACAGCATCGGCCTGGACATCGGCACCAACTCTGTGGGCTGGGCCGTGATCACCGACGAGTACAAGGTGCCCAGCAAGAAATTCAAGGTGCTGGGCAACACCGACCGGCACAGCATCAAGAAGAACCTGATCGGAGCCCTGCTGTTCGACAGCGGCGAAACAGCCGAGGCCACCCGGCTGAAGAGAACCGCCAGAAGAAGATACACCAGACGGAAGAACCGGATCTGCTATCTGCAAGAGATCTTCAGCAACGAGATGGCCAAGGTGGACGACAGCTTCTTCCACAGACTGGAAGAGTCCTTCCTGGTGGAAGAGGATAAGAAGCACGAGCGGCACCCCATCTTCGGCAACATCGTGGACGAGGTGGCCTACCACGAGAAGTACCCCACCATCTACCACCTGAGAAAGAAACTGGTGGACAGCACCGACAAGGCCGACCTGCGGCTGATCTATCTGGCCCTGGCCCACATGATCAAGTTCCGGGGCCACTTCCTGATCGAGGGCGACCTGAACCCCGACAACAGCGACGTGGACAAGCTGTTCATCCAGCTGGTGCAGACCTACAACCAGCTGTTCGAGGAAAACCCCATCAACGCCAGCGGCGTGGACGCCAAGGCCATCCTGTCTGCCAGACTGAGCAAGAGCAGACGGCTGGAAAATCTGATCGCCCAGCTGCCCGGCGAGAAGAAGAATGGCCTGTTCGGAAACCTGATTGCCCTGAGCCTGGGCCTGACCCCCAACTTCAAGAGCAACTTCGACCTGGCCGAGGATGCCAAACTGCAGCTGAGCAAGGACACCTACGACGACGACCTGGACAACCTGCTGGCCCAGATCGGCGACCAGTACGCCGACCTGTTTCTGGCCGCCAAGAACCTGTCCGACGCCATCCTGCTGAGCGACATCCTGAGAGTGAACACCGAGATCACCAAGGCCCCCCTGAGCGCCTCTATGATCAAGAGATACGACGAGCACCACCAGGACCTGACCCTGCTGAAAGCTCTCGTGCGGCAGCAGCTGCCTGAGAAGTACAAAGAGATTTTCTTCGACCAGAGCAAGAACGGCTACGCCGGCTACATTGACGGCGGAGCCAGCCAGGAAGAGTTCTACAAGTTCATCAAGCCCATCCTGGAAAAGATGGACGGCACCGAGGAACTGCTCGTGAAGCTGAACAGAGAGGACCTGCTGCGGAAGCAGCGGACCTTCGACAACGGCAGCATCCCCCACCAGATCCACCTGGGAGAGCTGCACGCCATTCTGCGGCGGCAGGAAGATTTTTACCCATTCCTGAAGGACAACCGGGAAAAGATCGAGAAGATCCTGACCTTCCGCATCCCCTACTACGTGGGCCCTCTGGCCAGGGGAAACAGCAGATTCGCCTGGATGACCAGAAAGAGCGAGGAAACCATCACCCCCTGGAACTTCGAGGAAGTGGTGGACAAGGGCGCTTCCGCCCAGAGCTTCATCGAGCGGATGACCAACTTCGATAAGAACCTGCCCAACGAGAAGGTGCTGCCCAAGCACAGCCTGCTGTACGAGTACTTCACCGTGTATAACGAGCTGACCAAAGTGAAATACGTGACCGAGGGAATGAGAAAGCCCGCCTTCCTGAGCGGCGAGCAGAAAAAGGCCATCGTGGACCTGCTGTTCAAGACCAACCGGAAAGTGACCGTGAAGCAGCTGAAAGAGGACTACTTCAAGAAAATCGAGTGCTTCGACTCCGTGGAAATCTCCGGCGTGGAAGATCGGTTCAACGCCTCCCTGGGCACATACCACGATCTGCTGAAAATTATCAAGGACAAGGACTTCCTGGACAATGAGGAAAACGAGGACATTCTGGAAGATATCGTGCTGACCCTGACACTGTTTGAGGACAGAGAGATGATCGAGGAACGGCTGAAAACCTATGCCCACCTGTTCGACGACAAAGTGATGAAGCAGCTGAAGCGGCGGAGATACACCGGCTGGGGCAGGCTGAGCCGGAAGCTGATCAACGGCATCCGGGACAAGCAGTCCGGCAAGACAATCCTGGATTTCCTGAAGTCCGACGGCTTCGCCAACAGAAACTTCATGCAGCTGATCCACGACGACAGCCTGACCTTTAAAGAGGACATCCAGAAAGCCCAGGTGTCCGGCCAGGGCGATAGCCTGCACGAGCACATTGCCAATCTGGCCGGCAGCCCCGCCATTAAGAAGGGCATCCTGCAGACAGTGAAGGTGGTGGACGAGCTCGTGAAAGTGATGGGCCGGCACAAGCCCGAGAACATCGTGATCGAAATGGCCAGAGAGAACCAGACCACCCAGAAGGGACAGAAGAACAGCCGCGAGAGAATGAAGCGGATCGAAGAGGGCATCAAAGAGCTGGGCAGCCAGATCCTGAAAGAACACCCCGTGGAAAACACCCAGCTGCAGAACGAGAAGCTGTACCTGTACTACCTGCAGAATGGGCGGGATATGTACGTGGACCAGGAACTGGACATCAACCGGCTGTCCGACTACGATGTGGACCATATCGTGCCTCAGAGCTTTCTGAAGGACGACTCCATCGACAACAAGGTGCTGACCAGAAGCGACAAGAACCGGGGCAAGAGCGACAACGTGCCCTCCGAAGAGGTCGTGAAGAAGATGAAGAACTACTGGCGGCAGCTGCTGAACGCCAAGCTGATTACCCAGAGAAAGTTCGACAATCTGACCAAGGCCGAGAGAGGCGGCCTGAGCGAACTGGATAAGGCCGGCTTCATCAAGAGACAGCTGGTGGAAACCCGGCAGATCACAAAGCACGTGGCACAGATCCTGGACTCCCGGATGAACACTAAGTACGACGAGAATGACAAGCTGATCCGGGAAGTGAAAGTGATCACCCTGAAGTCCAAGCTGGTGTCCGATTTCCGGAAGGATTTCCAGTTTTACAAAGTGCGCGAGATCAACAACTACCACCACGCCCACGACGCCTACCTGAACGCCGTCGTGGGAACCGCCCTGATCAAAAAGTACCCTAAGCTGGAAAGCGAGTTCGTGTACGGCGACTACAAGGTGTACGACGTGCGGAAGATGATCGCCAAGAGCGAGCAGGAAATCGGCAAGGCTACCGCCAAGTACTTCTTCTACAGCAACATCATGAACTTTTTCAAGACCGAGATTACCCTGGCCAACGGCGAGATCCGGAAGCGGCCTCTGATCGAGACAAACGGCGAAACCGGGGAGATCGTGTGGGATAAGGGCCGGGATTTTGCCACCGTGCGGAAAGTGCTGAGCATGCCCCAAGTGAATATCGTGAAAAAGACCGAGGTGCAGACAGGCGGCTTCAGCAAAGAGTCTATCCTGCCCAAGAGGAACAGCGATAAGCTGATCGCCAGAAAGAAGGACTGGGACCCTAAGAAGTACGGCGGCTTCGACAGCCCCACCGTGGCCTATTCTGTGCTGGTGGTGGCCAAAGTGGAAAAGGGCAAGTCCAAGAAACTGAAGAGTGTGAAAGAGCTGCTGGGGATCACCATCATGGAAAGAAGCAGCTTCGAGAAGAATCCCATCGACTTTCTGGAAGCCAAGGGCTACAAAGAAGTGAAAAAGGACCTGATCATCAAGCTGCCTAAGTACTCCCTGTTCGAGCTGGAAAACGGCCGGAAGAGAATGCTGGCCTCTGCCGGCGAACTGCAGAAGGGAAACGAACTGGCCCTGCCCTCCAAATATGTGAACTTCCTGTACCTGGCCAGCCACTATGAGAAGCTGAAGGGCTCCCCCGAGGATAATGAGCAGAAACAGCTGTTTGTGGAACAGCACAAGCACTACCTGGACGAGATCATCGAGCAGATCAGCGAGTTCTCCAAGAGAGTGATCCTGGCCGACGCTAATCTGGACAAAGTGCTGTCCGCCTACAACAAGCACCGGGATAAGCCCATCAGAGAGCAGGCCGAGAATATCATCCACCTGTTTACCCTGACCAATCTGGGAGCCCCTGCCGCCTTCAAGTACTTTGACACCACCATCGACCGGAAGAGGTACACCAGCACCAAAGAGGTGCTGGACGCCACCCTGATCCACCAGAGCATCACCGGCCTGTACGAGACACGGATCGACCTGTCTCAGCTGGGAGGCGACAAAAGGCCGGCGGCCACGAAAAAGGCCGGCCAGGCAAAAAAGAAAAAGGATCCACTTAACGTTACTGAAATCATCAAACAGCTTGACGAATCTGGATATAAGATCGTTGGTGTCGATGTCAGCTCCGGAGTTGAGACAAATGGTGTTCAGGATCTCGATAAGATACGTTCATTTGTCCAAGCAGCAAAGAGTGCCTTCTAGTGATTTAATAGCTCCATGTCAACAAGAATAAAACGCGTTTTCGGGTTTACCTCTTCCAGATACAGCTCATCTGCAATGCATTAATGCATTGACTGCAACCTAGTAACGCCTTNCAGGCTCCGGCGAAGAGAAGAATAGCTTAGCAGAGCTATTTTCATTTTCGGGAGACGAGATCAAGCAGATCAACGGTCGTCAAGAGACCTACGAGACTGAGGAATCCGCTCTTGGCTCCACGCGACTATATATTTGTCTCTAATTGTACTTTGACATGCTCCTCTTCTTTACTCTGATAGCTTGACTATGAAAATTCCGTCACCAGCNCCTGGGTTCGCAAAGATAATTGCATGTTTCTTCCTTGAACTCTCAAGCCTACAGGACACACATTCATCGTAGGTATAAACCTCGAAATCANTTCCTACTAAGATGGTATACAATAGTAACCATGCATGGTTGCCTAGTGAATGCTCCGTAACACCCAATACGCCGGCCGAAACTTTTTTACAACTCTCCTATGAGTCGTTTACCCAGAATGCACAGGTACACTTGTTTAGAGGTAATCCTTCTTTCTAGAAGTCCTCGTGTACTGTGTAAGCGCCCACTCCACATCTCCACTCGACCTGCAGGCATGCAGAATTCCCTTGTATCTCTACACACAGGCTCAAATCAATAAGAAGAACGGTTCGTCTTTTTCGTTTATATCTTGCATCGTCCCAAAGCTATTGGCGGGATATTCTGTTTGCAGTTGGCTGACTTGAAGTAATCTCTGCAGATCTTTCGACACTGAAATACGTCGAGCCTGCTCCGCTTGGAAGCGGCGAGGAGCCTCGTCCTGTCACAACTACCAACATGGAGTACGATAAGGGCCAGTTCCGCCAGCTCATTAAGAGCCAGTTCATGGGCGTTGGCATGATGGCCGTCATGCATCTGTACTTCAAGTACACCAACGCTCTTCTGATCCAGTCGATCATCCGCTGAAGGCGCTTTCGAATCTGGTTAAGATCCACGTCTTCGGGAAGCCAGCGACTGGTGACCTCCAGCGTCCCTTTAAGGCTGCCAACAGCTTTCTCAGCCAGGGCCAGCCCAAGACCGACAAGGCCTCCCTCCAGAACGCCGAGAAGAACTGGAGGGGTGGTGTCAAGGAGGAGTAAGCTCCTTATTGAAGTCGGAGGACGGAGCGGTGTCAAGAGGATATTCTTCGACTCTGTATTATAGATAAGATGATGAGGAATTGGAGGTAGCATAGCTTCATTTGGATTTGCTTTCCAGGCTGAGACTCTAGCTTGGAGCATAGAGGGTCCTTTGGCTTTCAATATTCTCAAGTATCTCGAGTTTGAACTTATTCCCTGTGAACCTTTTATTCACCAATGAGCATTGGAATGAACATGAATCTGAGGACTGCAATCGCCATGAGGTTTTCGAAATACATCCGGATGTCGAAGGCTTGGGGCACCTGCGTTGGTTGAATTTAGAACGTGGCACTATTGATCATCCGATAGCTCTGCAAAGGGCGTTGCACAATGCAAGTCAAACGTTGCTAGCAGTTCCAGGTGGAATGTTATGATGAGCATTGTATTAAATCAGGAGATATAGCATGATCTCTAGTTAGCTCACCACAAAAGTCAGACGGCGTAACCAAAAGTCACACAACACAAGCTGTAAGGATTTCGGCACGGCTACGGAAGACGGAGAAGCCACCTTCAGTGGACTCGAGTACCATTTAATTCTATTTGTGTTTGATCGAGACCTAATACAGCCCCTACAACGACCATCAAAGTCGTATAGCTACCAGTGAGGAAGTGGACTCAAATCGACTTCAGCAACATCTCCTGGATAAACTTTAAGCCTAAACTATACAGAATAAGATAGGTGGAGAGCTTATACCGAGCTCCCAAATCTGTCCAGATCATGGTTGACCGGTGCCTGGATCTTCCTATAGAATCATCCTTATTCGTTGACCTAGCTGATTCTGGAGTGACCCAGAGGGTCATGACTTGAGCCTAAAATCCGCCGCCTCCACCATTTGTAGAAAAATGTGACGAACTCGTGAGCTCTGTACAGTGACCGGTGACCGGAGAGACGGACGGACGCAGAGAGAAGGGCTGAGTAATAAGCCACTGGCCAGACAGCTCTGGCGGCTCTGAGGTGCAGTGGATGATTATTAATCCGGGACCGGCCGCCCCTCCGCCCCGAAGTGGAAAGGCTGGTGTGCCCCTCGTTGACCAAGAATCTATTGCATCATCGGAGAATATGGAGCTTCATCGAATCACCGGCAGTAAGCGAAGGAGAATGTGAAGCCAGGGGTGTATAGCCGTCGGCGAAATAGCATGCCATTAACCTAGGTACAGAAGTCCAATTGCTTCCGATCTGGTAAAAGATTCACGAGATAGTACCTTCTCCGAAGTAGGTAGAGCGAGTACCCGGCGCGTAAGCTCCCTAATTGGCCCATCCGGCATCTGTAGGGCGTCCAAATATCGTGCCTCTCCTGCTTTGCCCGGTGTATGAAACCGGAAAGGCCGCTCAGGAGCTGGCCAGCGGCGCAGACCGGGAACACAAGCTGGCAGTCGACCCATCCGGTGCTCTGCACTCGACCTGCTGAGGTCCCTCAGTCCCTGGTAGGCAGCTTTGCCCCGTCTGTCCGCCCGGTGTGTCGGCGGGGTTGACAAGGTCGTTGCGTCAGTCCAACATTTGTTGCCATATTTTCCTGCTCTCCCCACCAGCTGCTCTTTTCTTTTCTCTTTCTTTTCCCATCTTCAGTATATTCATCTTCCCATCCAAGAACCTTTATTTCCCCTAAGTAAGTACTTTGCTACATCCATACTCCATCCTTCCCATCCCTTATTCCTTTGAACCTTTCAGTTCGAGCTTTCCCACTTCATCGCAGCTTGACTAACAGCTACCCCGCTTGAGCAGACATCACCATGCCTGAACTCACCGCGACGTATGACCCAATACACCGCTGATGAGTCCGTGAGGACGAAACGAGTAAGCTCGTCCGGTGTTGAACTTCTCGCCGGTTTTAGAGCTAGAAATAGCAAGTTAAAATAAGGCTAGTCCGTTATCAACTTGAAAAAGTGGCACCGAGTCGGTGCTTTTGGCCGGCATGGTCCCAGCCTCCTCGCTGGCGCCGGCTGGGCAACATGCTTCGGCATGGCGAATGGGACATTGGGTCCACTTAACGTTACTGAAATCATCAAACAGCTTGACGAATCTGGATATAAGATCGTTGGTGTCGATGTCAGCTCCGGAGTTGAGACAAATGGTGTTCAGGATCTCGATAAGATACGTTCATTTGTCCAAGCAGCAAAGAGTGCCTTCTAGTGATTTAATAGCTCCATGTCAACAAGAATAAAACGCGTTTTCGGGTTTACCTCTTCCAGATACAGCTCATCTGCAATGCATTAATGCATTGACTGCAACCTAGTAACGCCTTNCAGGCTCCGGCGAAGAGAAGAATAGCTTAGCAGAGCTATTTTCATTTTCGGGAGACGAGATCAAGCAGATCAACGGTCGTCAAGAGACCTACGAGACTGAGGAATCCGCTCTTGGCTCCACGCGACTATATATTTGTCTCTAATTGTACTTTGACATGCTCCTCTTCTTTACTCTGATAGCTTGACTATGAAAATTCCGTCACCAGCNCCTGGGTTCGCAAAGATAATTGCATGTTTCTTCCTTGAACTCTCAAGCCTACAGGACACACATTCATCGTAGGTATAAACCTCGAAATCANTTCCTACTAAGATGGTATACAATAGTAACCATGCATGGTTGCCTAGTGAATGCTCCGTAACACCCAATACGCCGGCCGAAACTTTTTTACAACTCTCCTATGAGTCGTTTACCCAGAATGCACAGGTACACTTGTTTAGAGGTAATCCTTCTTTCTAGAAGTCCTCGTGTACTGTGTAAGCGCTCCGTCTCCATTGGCTCTTGGCAATTCGGTCAGCGGGGCTGACTGCCTCAGGTGGGGCAGTGCTAGTGTGTGTACCGACCCGCAGGATTGGTGCTTTGCCCAGAGCTCTACAGAATAGCGCGCGCATCCATATGTTAGTTCTGCAATTTTCTTGTATCGGTGCTGTGACTCATACTTCCCCCTTTGGCTGGCCTTGCGGCAACCAATAAGAACGCACAGTGAAATCTTGCGGGTGGGGAGTGGATCCATGGCGCCTGCATTGGCTTGGGGACGCGCACTGTCGCACACTTCCATCTGACCTTTCAGAAGGGTTTCGTGGTGGGCAAGGACCAACCGGTTGCGCGGCCGTGCGTGGGTGCCTCGCCCGGCACTGCCAGGGCCACTGCAGTGGCAGTTTGCTGCCTGATACAAAATCCTTCCCTCCGCCCAGTTTTCCCTCTTTGACCTTCCTTTCTCTTCTCTGCAACCAAATCCACCCTATCAAACCAAAACAGTATCTCGACCGAGGTATCAACCTGAATCAGCAACATCGTAGCCAGCATTTGTCTCCGTCTCTGCAGAACCAGCGAGTTGCAAACATTATCCAGGCAACAGGGCACCAACTCACTTCTTCGGCTTTCACCAATCGGTACAGCTCTTCTCAGAACTCGCGTCCGCAACAGTTCTACGCTTCCTCAGCACCTTCTTCAGCTTCAATCCTGAACACTCAGAACCGCGCACAGCAGCGCCCTCCTGTTCCCTTGTTTCCCAAAAGTACCGGTAGTATTTCGCACGGAAAGCAGGGCAACAAGATGTTCTCAGGTACCCATATGAAAAAGCCTGAACTCACCGCGACGTCTGTCGAGAAGTTTCTGATCGAAAAGTTCGACAGCGTCTCCGACCTGATGCAGCTCTCGGAGGGCGAAGAATCTCGTGCTTTCAGCTTCGATGTAGGAGGGCGTGGATATGTCCTGCGGGTAAATAGCTGCGCCGATGGTTTCTACAAAGATCGTTATGTTTATCGGCACTTTGCATCGGCCGCGCTCCCGATTCCGGAAGTGCTTGACATTGGGGAATTCAGCGAGAGCCTGACCTATTGCATCTCCCGCCGTGCACAGGGTGTCACGTTGCAAGACCTGCCTGAAACCGAACTGCCCGCTGTTCTGCAGCCGGTCGCGGAGGCCATGGATGCGATCGCTGCGGCCGATCTTAGCCAGACGAGCGGGTTCGGCCCATTCGGACCGCAAGGAATCGGTCAATACACTACATGGCGTGATTTCATATGCGCGATTGCTGATCCCCATGTGTATCACTGGCAAACTGTGATGGACGACACCGTCAGTGCGTCCGTCGCGCAGGCTCTCGATGAGCTGATGCTTTGGGCCGAGGACTGCCCCGAAGTCCGGCACCTCGTGCACGCGGATTTCGGCTCCAACAATGTCCTGACGGACAATGGCCGCATAACAGCGGTCATTGACTGGAGCGAGGCGATGTTCGGGGATTCCCAATACGAGGTCGCCAACATCTTCTTCTGGAGGCCGTGGTTGGCTTGTATGGAGCAGCAGACGCGCTACTTCGAGCGGAGGCATCCGGAGCTTGCAGGATCGCCGCGGCTCCGGGCGTATATGCTCCGCATTGGTCTTGACCAACTCTATCAGAGCTTGGTTGACGGCAATTTCGATGATGCAGCTTGGGCGCAGGGTCGATGCGACGCAATCGTCCGATCCGGAGCCGGGACTGTCGGGCGTACACAAATCGCCCGCAGAAGCGCGGCCGTCTGGACCGATGGCTGTGTAGAAGTACTCGCCGATAGTGGAAACCGACGCCCCAGCACTCGTCCGAGGGCAAAGGAATAGAGTAGATGCCGACCGGGATCCACTTAACGTTACTGAAATCATCAAACAGCTTGACGAATCTGGATATAAGATCGTTGGTGTCGATGTCAGCTCCGGAGTTGAGACAAATGGTGTTCAGGATCTCGATAAGATACGTTCATTTGTCCAAGCAGCAAAGAGTGCCTTCTAGTGATTTAATAGCTCCATGTCAACAAGAATAAAACGCGTTTCGGGTTTACCTCTTCCAGATACAGCTCATCTGCAATGCATTAATGCATTGGACCTCGCAACCCTAGTACGCCCTTCAGGCTCCGGCGAAGCAGAAGAATAGCTTAGCAGAGTCTATTTTCATTTTCGGGAGACGAGATCAAGCAGATCAACGGTCGTCAAGAGACCTACGAGACTGAGGAATCCGCTCTTGGCTCCACGCGACTATATATTTGTCTCTAATTGTACTTTGACATGCTCCTCTTCTTTACTCTGATAGCTTGACTATGAAAATTCCGTCACCAGCCCCTGGGTTCGCAAAGATAATTGCACTGTTTCTTCCTTGAACTCTCAAGCCTACAGGACACACATTCATCGTAGGTATAAACCTCGAAAATCATTCCTACTAAGATGGGTATACAATAGTAACCATGGTTGCCTAGTGAATGCTCCGTAACACCCAATACGCCGGCCGAAACTTTTTTACAACTCTCCTATGAGTCGTTTACCCAGAATGCACAGGTACACTTGTTTAGAGGTAATCCTTCTTTCTAGCTAGAGGATCCTCTACGCCGGACGCATCGTGGCCGGCATCACCGGCGCCACAGGTGCGGTTGCTGGCGCCTATATCGCCGACATCACCGATGGGGAAGATCGGGCTCGCCACTTCGGGCTCATGAGCGCTTGTTTCGGCGTGGGTATGGTGGCAGGCCCCGTGGCCGGGGGACTGTTGGGCGCCATCTCCTTGCTGCACTCTCAGTACAATCTGCTCTGATGCCGCATAGTTAAGCCAGCCCCGACACCCGCCAACACCCGCTGACGCGCCCTGACGGGCTTGTCTGCTCCCGGCATCCGCTTACAGACAAGCTGTGACCGTCTCCGGGAGCTGCATGTGTCAGAGGTTTTCACCGTCATCACCGAAACGCGCGAGACGAAAGGGCCTCGTGATACGCCTATTTTTATAGGTTAATGTCATGATAATAATGGTTTCTTAGACGTCAGGTGGCACTTTTCGGGGAAATGTGCGCGGAACCCCTATTTGTTTATTTTTCTAAATACATTCAAATATGTATCCGCTCATGAGACAATAACCCTGATAAATGCTTCAATAATATTGAAAAAGGAAGAGTATGAGTATTCAACATTTCCGTGTCGCCCTTATTCCCTTTTTTGCGGCATTTTGCCTTCCTGTTTTTGCTCACCCAGAAACGCTGGTGAAAGTAAAAGATGCTGAAGATCAGTTGGGTGCACGAGTGGGTTACATCGAACTGGATCTCAACAGCGGTAAGATCCTTGAGAGTTTTCGCCCCGAAGAACGTTTTCCAATGATGAGCACTTTTAAAGTTCTGCTATGTGGCGCGGTATTATCCCGTATTGACGCCGGGCAAGAGCAACTCGGTCGCCGCATACACTATTCTCAGAATGACTTGGTTGAGTACTCACCAGTCACAGAAAAGCATCTTACGGATGGCATGACAGTAAGAGAATTATGCAGTGCTGCCATAACCATGAGTGATAACACTGCGGCCAACTTACTTCTGACAACGATCGGAGGACCGAAGGAGCTAACCGCTTTTTTGCACAACATGGGGGATCATGTAACTCGCCTTGATCGTTGGGAACCGGAGCTGAATGAAGCCATACCAAACGACGAGCGTGACACCACGATGCCTGTAGCAATGGCAACAACGTTGCGCAAACTATTAACTGGCGAACTACTTACTCTAGCTTCCCGGCAACAATTAATAGACTGGATGGAGGCGGATAAAGTTGCAGGACCACTTCTGCGCTCGGCCCTTCCGGCTGGCTGGTTTATTGCTGATAAATCTGGAGCCGGTGAGCGTGGGTCTCGCGGTATCATTGCAGCACTGGGGCCAGATGGTAAGCCCTCCCGTATCGTAGTTATCTACACGACGGGGAGTCAGGCAACTATGGATGAACGAAATAGACAGATCGCTGAGATAGGTGCCTCACTGATTAAGCATTGGTAACTGTCAGACCAAGTTTACTCATATATACTTTAGATTGATTTAAAACTTCATTTTTAATTTAAAAGGATCTAGGTGAAGATCCTTTTTGATAATCTCATGACCAAAATCCCTTAACGTGAGTTTTCGTTCCACTGAGCGTCAGACCCCGTAGAAAAGATCAAAGGATCTTCTTGAGATCCTTTTTTTCTGCGCGTAATCTGCTGCTTGCAAACAAAAAAACCACCGCTACCAGCGGTGGTTTGTTTGCCGGATCAAGAGCTACCAACTCTTTTTCCGAAGGTAACTGGCTTCAGCAGAGCGCAGATACCAAATACTGTCCTTCTAGTGTAGCCGTAGTTAGGCCACCACTTCAAGAACTCTGTAGCACCGCCTACATACCTCGCTCTGCTAATCCTGTTACCAGTGGCTGCTGCCAGTGGCGATAAGTCGTGTCTTACCGGGTTGGACTCAAGACGATAGTTACCGGATAAGGCGCAGCGGTCGGGCTGAACGGGGGGTTCGTGCACACAGCCCAGCTTGGAGCGAACGACCTACACCGAACTGAGATACCTACAGCGTGAGCTATGAGAAAGCGCCACGCTTCCCGAAGGGAGAAAGGCGGACAGGTATCCGGTAAGCGGCAGGGTCGGAACAGGAGAGCGCACGAGGGAGCTTCCAGGGGGAAACGCCTGGTATCTTTATAGTCCTGTCGGGTTTCGCCACCTCTGACTTGAGCGTCGATTTTTGTGATGCTCGTCAGGGGGGCGGAGCCTATGGAAAAACGCCAGCAACGCGGCCTTTTTACGGTTCCTGGCCTTTTGCTGGCCTTTTGCTCACATGTTCTTTCCTGCGTTATCCCCTGATTCTGTGGATAACCGTATTACCGCCTTTGAGTGAGCTGATACCGCTCGCCGCAGCCGAACGACCGAGCGCAGCGAGTCAGTGAGCGAGGAAGCGGAAGAGCGCCCAATACGCAAACCGCCTCTCCCCGCGCGTTGGCCGATTCATTAATGCAGCTGGCACGACAGGTTTCCCGACTGGAAAGCGGGCAGTGAGCGCAACGCAATTAATGTGAGTTAGCTCACTCATTAGGCACCCCAGGCTTTACACTTTATGCTTCCGGCTCGTATGTTGTGTGGAATTGTGAGCGGATAACAATTTCACACAGGAAACAGCTATGACCATGATTAC |
